# *De novo* functional protein sequence generation: overcoming data scarcity through regeneration and large language models

**DOI:** 10.1093/bib/bbag095

**Published:** 2026-03-08

**Authors:** Chenyu Ren, Daihai He, Jian Huang

**Affiliations:** Department of Applied Mathematics, The Hong Kong Polytechnic University, Hung Hom, Kowloon, Hong Kong, China; Department of Applied Mathematics, The Hong Kong Polytechnic University, Hung Hom, Kowloon, Hong Kong, China; Department of Applied Mathematics, The Hong Kong Polytechnic University, Hung Hom, Kowloon, Hong Kong, China; Department of Data Science and AI, The Hong Kong Polytechnic University, Hung Hom, Kowloon, Hong Kong, China

**Keywords:** generative models, large language models, protein design, representation learning, small sample size

## Abstract

Proteins are essential components of all living organisms and play a critical role in cellular survival. They have a broad range of applications, from clinical treatments to material engineering. This versatility has spurred the development of protein design, with amino acid sequence design being a crucial step in the process. Recent advancements in deep generative models have shown promise for protein sequence design. However, the scarcity of functional protein sequence data for certain types can hinder the training of these models, which often require large datasets. To address this challenge, we propose a hierarchical model named ProteinRG that can generate functional protein sequences using relatively small datasets. ProteinRG begins by generating a representation of a protein sequence, leveraging existing large protein sequence models, before producing a functional protein sequence. We have tested our model on various functional protein sequences and evaluated the results from three perspectives: multiple sequence alignment, t-SNE distribution analysis, and 3D structure prediction. The findings indicate that our generated protein sequences maintain both similarity to the original sequences and consistency with the desired functions. Moreover, our model demonstrates superior performance compared twith other generative models for protein sequence generation.

## Introduction

Functional proteins are essential components of all living organisms and play a critical role in cellular survival. They have a broad range of applications, from clinical treatments, such as targeting cancer cells and facilitating gene therapy, to material engineering, where enzymes are used to degrade plastics. Their significance has catalyzed advancements in *de novo* protein design, particularly in the creation of functional proteins. While traditional protein design methods have made significant progress over the past decades [1–3], they are not without their drawbacks. These methods often employ computational techniques like the Monte Carlo algorithm to deduce optimal sequences based on a given backbone scaffold, rather than specific functions. Such processes can be computationally intensive and time-consuming, especially when incorporating the functional attributes of proteins.

In contrast, deep learning has gained traction as a powerful and efficient approach to protein engineering. A notable example is AlphaFold [4], an AI model introduced in 2020, which has been recognized as a breakthrough in predicting protein 3D structures, effectively addressing the protein folding problem. Nonetheless, the challenge of *de novo* protein design persists; it involves designing the primary structure, or amino acid sequence, of a functional protein. This sequence is the starting point for models like AlphaFold, as it dictates the protein’s physicochemical properties, molecular function, and ultimately, its 3D structure.

The rapid advancements in generative models, such as generative adversarial networks (GAN) [5], variational autoencoders (VAE) [6], and diffusion models [7, 8], have revolutionized the fields of image and text generation. These models also hold significant promise for protein design, with various proposed models leveraging them to craft distinct functional amino acid sequences [9] introduced an unconditional GAN for generating the malate dehydrogenase (MDH) enzyme, and [10] employed a conditional GAN to create different functional proteins based on given Gene Ontology (GO) annotations.

However, a major challenge in the supervised training of these generative models is the scarcity of labeled data [11] highlighted the limited availability of unique hexon sequences, with only 711 unique full-length sequences from the UniprotKB database, which hampers efficient training. A similar issue arises in the design of lysozyme, an enzyme crucial for its antibacterial, anti-inflammatory, and antiviral properties in medicine, cosmetics, and food preservation [12–15]. Most natural lysozyme is derived from bird species, which poses a problem for individuals allergic to egg white [16, 17]. The UniProtKB database contains $\sim $2650 unique full-length sequences for Lysozyme, comprising 1163 sequences for Lysozyme C and 1490 sequences for Lysozyme G, and some lack essential keywords. This limited dataset poses a significant challenge for effectively training generative models. Another problem is how to obtain protein sequences with desired functions that could remove the effect of allergenicity.

The recent advent of ChatGPT has demonstrated the potential of large language models (LLMs), particularly through the application of representation learning and transfer learning. This approach involves pretraining on a vast corpus of unlabeled data followed by fine-tuning on a smaller set of labeled data, effectively addressing the challenge of data scarcity. The application of LLMs to protein sequences has gained considerable attention in recent years. AlphaFold [4], developed by DeepMind, uses deep learning and attention mechanisms to predict protein structures with high accuracy. ProtBert [18] trained on large protein sequence databases to perform various prediction tasks, such as the prediction of protein functions. Evolutionary scale modeling (ESM) [19] leverages transformer architectures to capture evolutionary patterns in protein sequences [20] developed an LLM capable of generating amino acid sequences based on specific keywords for various protein families. However, the reliance on keywords presents a challenge, as they are not always available or complete in the UniProtKB database. Additionally, retraining a pretrained model to accommodate different protein descriptions can be prohibitively expensive.

To address these challenges, we introduce ProteinRG, a hierarchical generative model inspired by regeneration learning [21]. Functional protein sequence design can be conceptualized as a task of conditional data generation, where the objective is to learn a mapping that can be used to sample new data from the conditional distribution of target data with a given condition. The concept of regeneration learning demonstrates significant promise in learning complex distributions. ProteinRG starts by generating a representation of a protein sequence, utilizing existing large protein sequence models. It then employs a conditional generative model, based on the representation and annotation, to produce a functional protein sequence. Our model can be trained on a limited dataset comprising various functional protein sequences. We evaluate the generated results from three distinct perspectives: multiple sequence alignment (MSA), t-SNE distribution analysis, and 3D structures corresponding to the sequences. Furthermore, we employ the maximum mean discrepancy (MMD) statistic to assess the distributional similarity and the mean reciprocal rank (MRR) to evaluate the conditional consistency of the generated protein sequences with the actual sequences. Our two-stage model demonstrates superior performance compared with other one-stage generative models for protein sequence design.

## Materials and methods

### Datasets

In the case study, we apply the proposed method to two protein sequence datasets available from the UniProt Knowledgebase [22]: the Lysozyme sequence dataset and the MDH sequence dataset. These datasets consist of protein sequences and protein keywords. They can be accessed online at https://www.uniprot.org/. Due to the occasional absence of protein keywords in the UniProtKB database, we have utilized GO annotations to ascertain the functions of protein sequences. The GO knowledge base is recognized globally as the definitive resource for the comprehensive description of gene functions. GO annotations provide insights into a gene’s molecular function, its cellular location, and the biological processes or pathways it is involved in. The details of the datasets are shown in the supplement.

#### Evaluation metrics

Since the primary objective of generative models is to accurately replicate the distribution of target data, it is logical to evaluate their performance using a two-sample statistic that compares the distributions of both the generated data and the training data. For protein sequence data, which pose challenges for direct analysis, this evaluation can be effectively conducted on extracted feature vectors. The MMD [23] serves as a test statistic for this purpose, comparing the mean embeddings within a reproducing kernel Hilbert space (RKHS).

In the context of conditional generation, it is crucial to evaluate the model’s ability to produce sequences that align with specific target labels. To this end, the MRR [10] enhances the MMD metric by calculating the MMD between subsets of sequences corresponding to each label. This involves ranking the RKHS distance between generated samples and their designated target label in comparison with distances from off-target labels. Essentially, this metric evaluates how frequently sets of real sequences with off-target labels are distributionally closer to the generated sequences than the real sequences bearing the target label. Also, we introduce the entropy and structure predictions to evaluate the novelty of generated protein sequences. The detailed definition of evaluation metrics is proposed in the supplementary.

### Method

**Figure 1:**
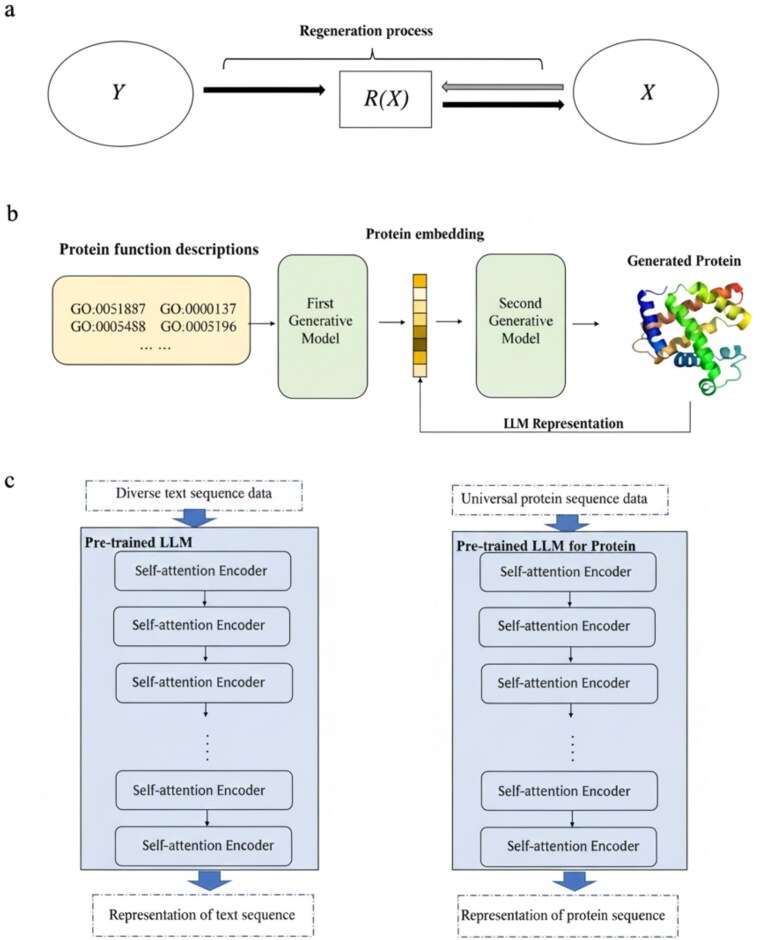
The concept of ProteinRG. a. The regeneration process is a concept designed for mapping complex distributions from $Y$ to $X$. The process begins by transforming $X$ into an abstract representation $R(X)$, followed by regenerating $R(X)$ from $Y$, and ultimately generating $X$ from $R(X)$. b. Our proposed ProteinRG for functional protein sequence generation adheres to this regeneration process. It commences with the generation of a representation of protein sequences, which is derived from a large protein sequence model, and subsequently generates the actual protein sequence. c. On the left, we have the pretrained LLM for natural language processing, while on the right is the pretrained large model specifically for proteins. Both models utilize multiple self-attention encoder blocks from the transformer architecture. The concept of ProteinRG illustrates a regeneration process in which abstract representations derived from large pretrained models—natural language on the left and proteins on the right, both built on transformer encoder blocks—are sequentially transformed and regenerated to produce functional protein sequences.

Let $X$ denote an amino acid sequence and $Y$ the corresponding annotation label or sequence type. ProteinRG aims to learn a generative model that enables sampling from the conditional distribution $ P(X \mid Y).$ For a given sequence $ X, $ let $ R(X) $ be an intermediate abstraction of $ X. $ Compared with $ X, $  $ R(X) $ lies in a lower dimensional space, making it easier to model, while retaining more information relevant to conditional generation than $ Y. $ The overall regeneration and generative learning framework is illustrated in Fig. 1.

Suppose the sequence length is $ L, $ the number of amino acid types is $ A, $ and the dimension of $ Y $ is $ d. $ We use a one-hot vector of length $ A $ to represent each amino acid, so the dimension of $ X $ is $ L \times A. $ This problem poses two main challenges. First, the conditional distribution $ P(X \mid Y) $ is unknown, and we only observe samples from the joint distribution of $ (X, Y). $ Second, $ X $ is high-dimensional. For protein sequences, a typical length is $ L = 2048, $ and with $ A = 21 $ amino acid types, the dimension of $ X $ is $ 2048 \times 21 = 43\,008. $ Training a generative model in such a high-dimensional space with limited data is extremely difficult.

To address this, we propose a regeneration method that leverages a pretrained large protein sequence model to alleviate data scarcity. Specifically, we take $ R:= R(X) $ to be a deterministic function that maps $ X $ from the $ (L \times A) $ -dimensional space to an $ m $-dimensional space, with $ m \ll L \times A. $ This mapping is designed to reduce dimensionality while preserving essential information for generation. We then exploit a useful factorization of the conditional distribution using $ R $: 


(1) \begin{align*}& P(X \mid Y) = P(X, R \mid Y) = P(X \mid R, Y) P(R \mid Y),\end{align*}

where the first equality follows from the fact that $R=R(X)$ is a deterministic function of $X,$ and the second equality follows from the standard factorization of joint probabilities. This decomposition breaks the original high-dimensional conditional distribution into more tractable components.

Therefore, ProteinRG aims to learn $P(X\mid Y)$ in a hierarchical manner based on equation (1). As outlined in Steps 1 and 2, instead of modeling $ P(X\mid Y)$ directly, ProteinRG learns to generate from the two simpler conditional distributions on the right-hand side of equation (1). This hierarchical generative strategy is analogous to approaches used in text-conditional image generation [24].

**Figure 2:**
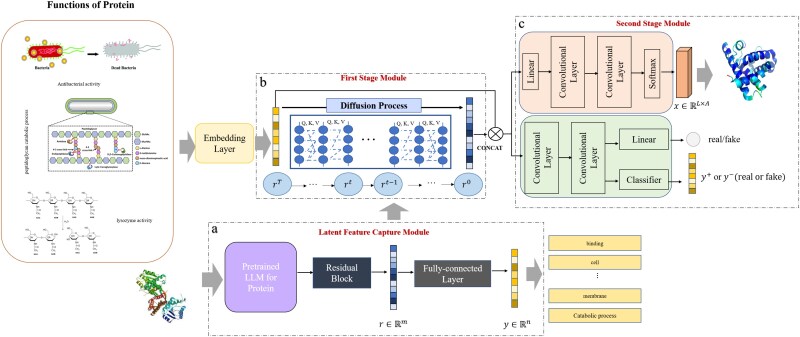
Overview of the proposed hierarchical functional protein generation model. Our model comprises three modules: (a) the latent feature capture module, (b) the first-stage generative module, and (c) the second-stage generative module. It begins by generating a latent representation of a protein sequence before producing a functional protein sequence.

An overview of ProteinRG is depicted in Fig. 2. The model comprises three modules that computationally implement the proposed method. The statistical basis of the method is expressed in equation (1).

- *Module 1: Protein sequence representation learning*: In this module, we fine-tune a pretrained large protein sequence model to learn a representation $R$ of a high-dimensional amino acid sequence $X$: $R = R(X)$.
- *Module 2: Learning the conditional distribution $P(R\mid Y)$*: In this module, we train a generative model $G_{1}$ to learn the conditional distribution $P(R \mid Y).$ This module is for generating latent representations for a given annotation label. That is, for a given label $Y=y$, this module allows us to sample $r \sim P(R\mid Y=y).$
- *Module 3: Learning the conditional distribution $P(X\mid R, Y)$*: In this module, we train a second generative model $G_{2}$ for learning the conditional distribution $P(X \mid R, Y)$. This module is for generating amino acid sequences conditional on $(R, Y)=(r, y)$: $x\sim P(X \mid R=r,Y= y),$ where $r$ is generated from the generative model in Module 2.

After we have trained the model as described above, the generating process is then as follows: for a given annotation $Y=y$,

- Step 1: generate $r \sim P(R\mid Y=y)$,
- Step 2: generate $x \sim P(X|R=r, Y=y).$

A key feature of our approach is that we fine-tune a pretrained large protein sequence model for learning the representation function $R,$ which captures more information than the annotation labels about the structural details of amino acid sequences. This makes the sampling process from $P(R|Y)$ and then from $P(X|R,Y)$ more effective than directly sampling from $P(X|Y)$. Our training dataset consists of pairs $\{x,y\} = \{ (x_{i},y_{i}): i = 1,2,...,N \}$, where $x_{i}$ represents the $i$th amino acid sequence, $y_{i}$ corresponds to its annotated label, and $N$ is the number of sequences.

Below we give more detailed descriptions of Modules 1–3.

In Module 1, we utilize ESM-2, a state-of-the-art LLM specifically designed for learning representations of proteins. This model has been pretrained with 15 billion parameters on a dataset comprising over 60 million protein sequences from the UniRef50 database, making it one of the largest protein language models evaluated to date. We fine-tune the ESM-2 model using training data. The downstream task is to classify protein functions. The model receives a set of protein sequences as input and produces multi-class GO annotations as output.

Specifically, we design a network architecture that includes a residual block and fully connected layers, tailored for the supervised learning task. We extract the latent representation of protein sequences from the residual block within the classification network. To fine-tune the pretrained large protein sequence model ESM-2, we use cross entropy (CE) as the loss function, which is defined as 


(2) \begin{align*}& L_{\mathrm{CE}}= -{E}_{(X,Y) \sim p(x,y)}\left[ \log p\left(Y \mid X\right)\right].\end{align*}

The training process involves adjusting the parameters of the classification network as well as certain parameters of the pretrained model. The remaining parameters in the ESM-2 model are kept fixed.

We note that using the output from the pretrained model directly, without passing it through the classification network, can also serve as a representation of the protein sequences. This is useful in a semi-supervised setting, where the dataset includes protein sequences whose annotations are not available.

In Module 2, we use a denoising diffusion probabilistic model (DDPM, [7]) and a transformer-based network [25] to learn a generative model for the conditional distribution $P(R \mid Y).$ This mode is used to generate the latent representations of protein sequences.

DDPM is a parameterized Markov chain trained using variational inference to generate samples that match the target data distribution within a finite number of steps [7]. Standard diffusion models incorporate two key phases: the forward process, which is used for training, and the reverse process, which is utilized for sampling. The critical aspect of training diffusion models is to estimate a denoising function for predicting noise that is added at each time step. The loss function for estimating the denoising function is expressed as a mean square error between the actual noise and the predicted noise. More details are given in the Supplementary Materials.

In our specific application of the diffusion model, we have found it more effective to predict the final latent representation $r$ directly, rather than predicting the noise in the forward process as in the original diffusion model. Therefore, we train the model using the following loss function, 


\begin{align*}& L_{\mathrm{diff}}(\theta) = {E}_{t \sim[1, T], R \sim q_{t}}\big[\big\|R_\theta (R, t, y)-r\big\|^{2}\big], \end{align*}

where $T$ represents the total number of diffusion time steps, $t$ is encoded using a cosine embedding, and $y$ is a given condition. The function $R_\theta $ is a decoder-only transformer [26] designed to generate the latent representation of a protein sequence, conditioned on the given annotation $y$.

To enhance the performance of sampling, we employ classifier-free guidance during training, which involves randomly omitting the condition $Y$ in 10% of the time steps. While the latent feature capture module constructs a link between the protein sequence $X$ and its latent representation $R$, the first stage generative module builds a mapping from functions of protein sequence $X$ to its latent representation $R$. We design a classifier-free guidance diffusion model [7] to generate the representation $R$ conditioned on the functions $Y$.

In Module 3, building upon the latent embedding $r$ obtained from Module 1 and the representation generated from Module 2, we use a Conditional Wasserstein Generative Adversarial Network (CWGAN) with Gradient Penalty (CWGAN-GP) [27] in the generation of protein sequences.

To enhance conditional consistency and address the issue of low intra-class diversity, we propose the integration of an auxiliary discriminative classifier capable of handling multiple classes. Drawing inspiration from ADCGAN [28], which introduces an auxiliary discriminative classifier to distinguish between real and generated data using binary labels, we adapt this approach to our multi-class labeled data. We employ mutually exclusive one-hot encoding for multi-class generated data, ensuring clear discrimination of the generated data from the real data.

The objective functions for the generator, the discriminator, and the discriminative classifier are described in detail in the Supplementary Materials.

## Results

In this section, we apply the proposed method to the mentioned protein sequence datasets. We evaluate the generated protein sequences from three distinct angles: at the 1D level using MSA, at the 2D level through t-SNE distribution analysis [29], and at the 3D level via AlphaFold2 [4]. The MSA is performed using Clustal Omega [30], and we further compute Shannon’s entropy for the MSA results at each position to assess sequence conservation. For dimensionality reduction, we employ the t-SNE technique, utilizing the scikit-learn t-SNE package with its default settings. We initially embed the protein sequences using ESM-2, which yields a 480D representation. These high-dimensional data are then condensed to a 2D space for visualization purposes. Additionally, MMseqs2 is utilized for clustering analysis at the 3D structural level.

### Lysozyme sequences

**Figure 3:**
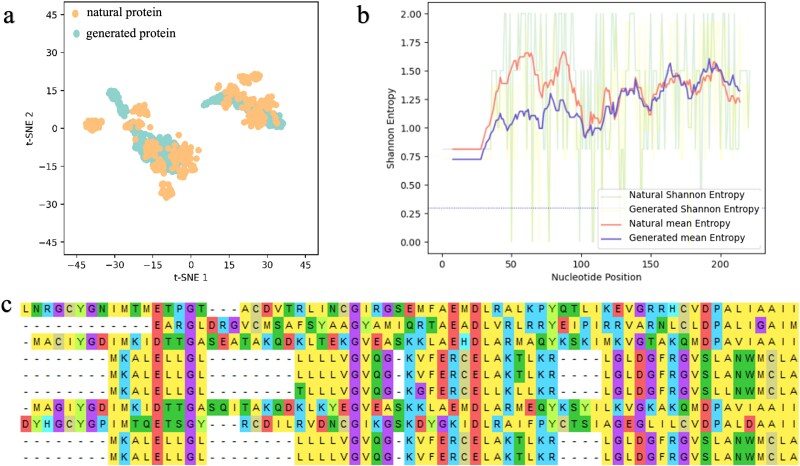
a.t-SNE visualization of natural protein from validation dataset and the generated protein. The top layer is natural protein sequences and the bottom layer is generated protein sequences. b. Shannon’s entropy at each position of the MSA results. c. MSA results of five generated lysozyme sequences and five natural lysozyme sequences randomly sampled from both lysozyme C and lysozyme G.

Generative models aim to capture the conditional distribution of real data. Figure 3a illustrates the 2D t-SNE visualization derived from a validation dataset comprising $\sim $500 natural lysozyme sequences, with an equivalent number of generated lysozyme sequences. The x-axis represents the first dimension of t-SNE, while the y-axis corresponds to the second dimension. In this visualization, the real distribution of natural lysozyme sequences is depicted in yellow, and the generated distribution of lysozyme sequences is shown in green.

For the t-SNE analysis, we enhance the representation of both real and generated data by employing the ESM-2 model to reduce the dimensionality of the data. We set the number of components, n_components, to 2, and retain the default settings for other parameters, which are unlikely to significantly affect the t-SNE results. Our findings indicate that the distribution of the generated lysozyme sequences closely resembles that of the real sequences, suggesting that the generative model has successfully learned the underlying distribution of the data.

Figure 3b presents the Shannon entropy calculated for each position in an MSA that includes six natural lysozyme sequences and six synthetically generated lysozyme sequences. To conduct this analysis, we utilized Clustal Omega to perform the MSA, incorporating both lysozyme C and lysozyme G for validation purposes. The outcomes of the MSA, depicted in Fig. 3c, suggest that the sequences are homologous. Further analysis involved calculating Shannon’s entropy for the MSA results at each position, which reflects the frequency or probabilities within the alignment. The entropy plot in Fig. 3b reveals that the entropy levels of both natural and generated sequences are comparable, indicating that our model is capable of learning the conditional distribution and retaining the characteristic features of lysozyme sequences.

**Figure 4:**
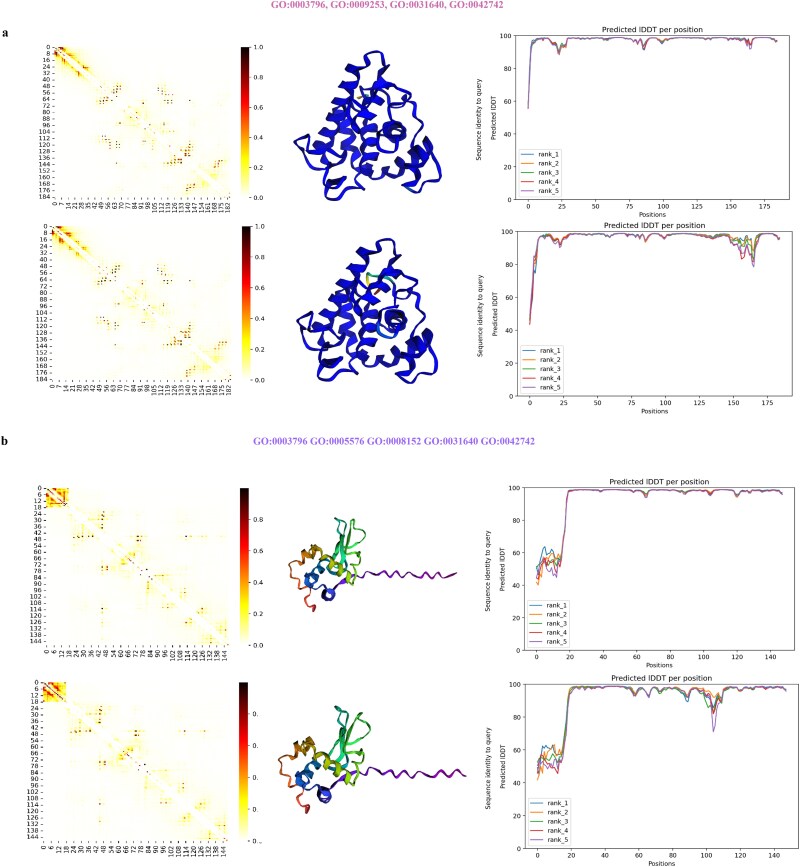
The analysis results of the natural lysozyme sequence (above) and the generated lysozyme sequence (below). The attention map, IDDT at each position, and 3D-structure prediction from Alphafold2 are given in **a** and **b**. a. GO:0003796, GO:0009253, GO:0031640, GO:0042742. b. GO:0003796 GO:0005576 GO:0008152 GO:0031640 GO:0042742.

The detailed predictions for the 2D and 3D structures of these sequences are analyzed in Fig. 4. In Fig. 4 (subfigures a, b), the results for the real lysozyme sequences are displayed above, while those for the generated sequences are below. Figure 4 presents the analysis of lysozyme functionality with different GO terms. We employed ESM-2 for predicting the 2D structures. The attention maps of two lysozyme sequences shown in Figs 4 illustrate the protein sequences’ structure through heatmaps, where darker colors indicate higher values, signifying contact between amino acid residues at specific positions. This contact information aids in inferring the 2D and 3D structures of the proteins. Figure 4 also displays the IDDT (Identification of Directly Determined Contacts [31]) results for each position, comparing the natural and generated sequences. The significance of these metrics is elaborated in the Supplementary Materials. From these results, it is evident that the generated lysozyme sequences retain most of the structural information. Lastly, Detailed 3D structure predictions by AlphaFold2 are shown in Fig. 4, where a visual comparison reveals a high degree of similarity between the generated and natural protein structures. Additional results are given in the Supplementary Materials.

### Malate dehydrogenase sequences

In our second example, we focus on MDH protein sequences. The process for training and validating the ProteinRG model with MDH sequences follows the same procedure as described for the Lysozyme sequences above.

**Figure 5:**
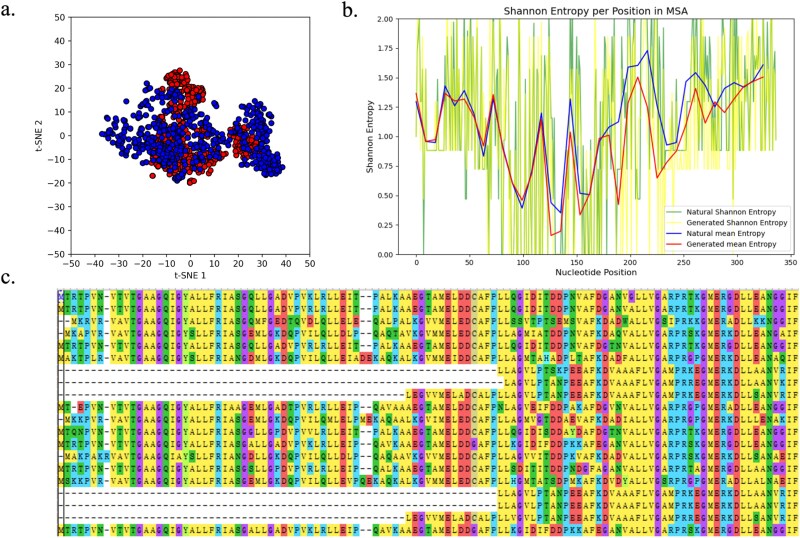
The general analysis results of the natural MDH sequence and the generated MDH sequence. a. The t-SNE result is about 500 natural MDH sequences and 500 generated MDH sequences. b. The Shannon entropy result with 10 natural MDH sequences and 10 generated MDH sequences. c. The MSA result is obtained by the MEGA software.

**Figure 6:**
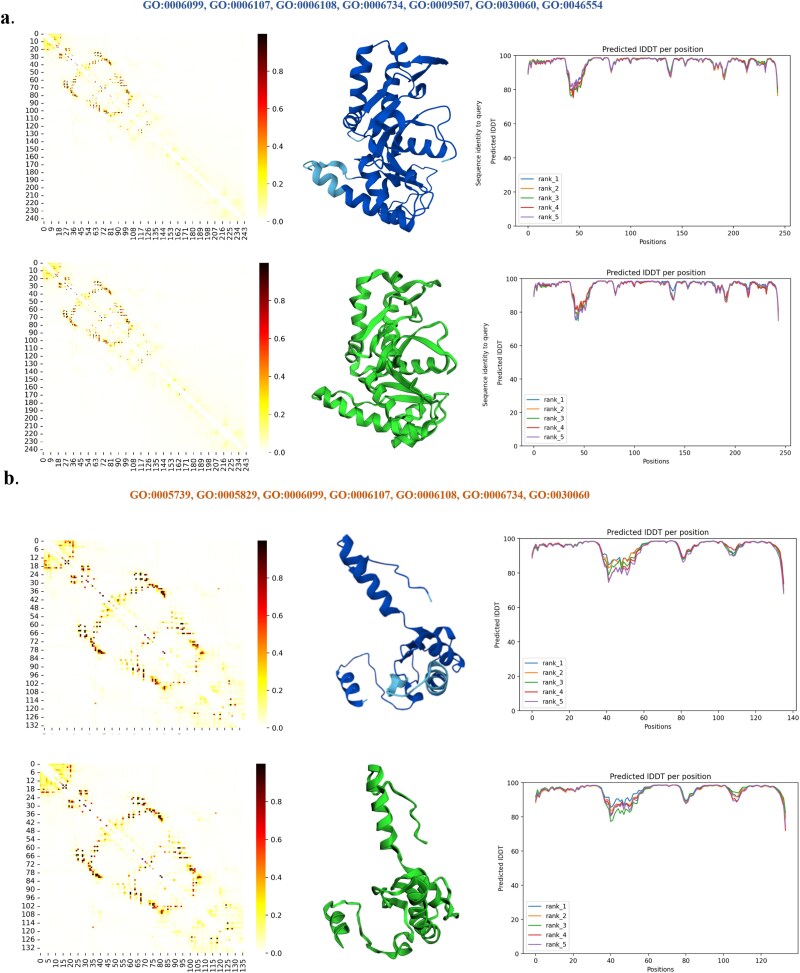
The analysis results of the natural MDH sequence (above) and the generated MDH sequence (below). The attention map, IDDT at each position, and 3D-structure prediction from Alphafold2 are given in **a** and **b**. **a**. The input GO annotations for the protein sequence include GO:0006099, 0006107, 0006108, 0006734, 0009507, 0030060, and 0046554. **b**. The input GO annotations include GO:0005739, 0005829, 0006099, 0006107, 0006108, 0006734, and 0030060.

Figure 5 show the general analysis result about the natural MDH sequences and generated MDH sequences, which contains t-SNE result in Fig. 5a, the Shannon entropy result with 20 sequences in Fig. 5b, and the MSA result in Fig. 5c. Figure 6a,b,c present the analysis results of the natural MDH sequence on the left and the generated MDH sequence on the right. The attention map that shows the 2D interactions between each amino acid of the sequence is predicted by ESM-2. The IDDT scores and 3D structure prediction are conducted by Alphafold2. The sequence conservation analysis is based on the MSA and MEGA for one natural MDH sequence and one generated MDH sequence with the same GO annotations.

Figures 6a and 6b present the analysis results, with the natural MDH sequence above and the generated MDH sequence below. The attention map, showing the 2D interactions between each amino acid of the sequence that is related to the contact map, is predicted by ESM-2. The IDDT scores and 3D structure predictions are conducted by AlphaFold2. For Fig. 6a, the input GO annotations include Tricarboxylic acid cycle (GO:0006099), Oxaloacetate metabolic process (GO:0006107), Malate metabolic process (GO:0006108), NADH metabolic process (GO:0006734), Chloroplast (GO:0009507), L-MDH activity (GO:0030060), MDH activity (GO:0046554). For Fig. 6b, the input GO annotations include Mitochondrion (GO:0005739), Cytosol (GO:0005829), Tricarboxylic acid cycle (GO:0006099), Oxaloacetate metabolic process (GO:0006107), Malate metabolic process (GO:0006108), NADH metabolic process (GO:0006734). For Fig. 6c, the input GO annotations include Tricarboxylic acid cycle (GO:0006099), Oxaloacetate metabolic process (GO:0006107), Malate metabolic process (GO:0006108), NADH metabolic process (GO:0006734).

Moreover, we present the sequence identity of the generated MDH sequences to the natural MDH sequences from the test dataset at different training steps in the Supplementary Materials. The number of selected generated sequences is 32. We calculate the MDH sequence identity using the Biopython package [32]. The final identity of the generated MDH sequences can reach up to 95%.

Regarding the novelty of generated protein sequences, we also quantify local novelty using a sliding-window percent identity to the nearest training sequence in the supplementary Figure S7b. This captures how similar each segment of the query is to known sequences. We used a sliding window of three residues. Combined with Shannon entropy, the analysis shows that positions with high identity and low entropy correspond to tightly constrained sites, whereas positions with lower identity and higher entropy mark flexible regions where diversity is tolerated and generated. Although the global sequence identity approaches 95%, local identity in specific regions can drop to below 50%.

We further assessed similarity between natural and generated proteins via structural alignment. Specifically, we computed TM-scores for four natural generated groups. As shown in Figure S6, the TM-scores indicate that the generated structures are largely consistent with their natural counterparts, preserving key functional motifs and overall bioactivity. In addition, we compared the stability of generated and natural sequences while explicitly incorporating prediction confidence as a quality-control criterion (Figure S7a in the supplement). Delta Delta G (DDG) is a metric for predicting how a single point mutation will affect protein stability. We visualized DDG with violin plots and conducted statistical tests on both the full dataset and a high-confidence subset (e.g. confidence $\ge 0.85$). Across conditions, the two groups exhibit largely overlapping DDG distributions with only small differences in mean and median.

### Comparative analysis: diverse protein sequences

We conducted a comparative analysis of our model against both LLMs for sequence data and deep-learning models for protein sequence generation.

We compare our method with the following methods.

- CVAE [33]: This model is a Conditional VAE designed for protein sequences. We adapted the model to accommodate the 50 labels pertinent to our problem setting and conducted a Bayesian optimization search for the best hyperparameters.
- ProGen [20]: ProGen is a language model leveraging a state-of-the-art Transformer architecture. Conditional information is integrated by prefixing label tokens to the sequence. We scaled down the model size and retrained it using our dataset.
- One-per-label GAN (OpL−GAN): OpL−GAN is an unconditional protein generation method. Therefore, in using this method, we consider one instance of ProteoGAN for each label, omitting the conditioning mechanism (resulting in a total of 50 models). Sequences for a specific label are generated by sampling from the GAN trained exclusively on sequences annotated with that label. This model allows us to evaluate whether training 50 distinct models could be an alternative to employing a conditioning mechanism.
- ProteoGAN [10]: A conditional GAN tailored for the functional design of proteins, utilizing hierarchical GO annotations. Unlike other models, ProteoGAN takes discrete labels as input rather than representations.

Since these methods are designed for protein sequence data with large sample sizes, they are not suitable for the Lysozyme and MDH datasets used in our analysis, as the sample sizes of these two datasets are too small for these existing methods to be effective. Therefore, we use the protein sequence dataset included in [10], which consists of 157 890 protein sequences, each annotated with one of 50 distinct GO functions.

The performance metrics—including MMD, MRR, average entropy difference (AED) between real and generated sequences, and distance between the distributions of real and generated sequences—are summarized in Table 1. The training process is illustrated in Figure S2 of the Supplementary Materials. Entropy is employed to assess protein sequence diversity by quantifying the variability at each position within a protein sequence alignment.

**Table 1:** Comparison of the performance of our model with the existing methods OpL−GAN, ProGen, CVAE, and ProteoGAN in terms of MMD (similarity), MRR (conditional consistency), AED (average entropy difference between real and generated sequences), and distance between the distributions of real and generated sequences.

Model	MMD (Similarity)	MRR (Conditional consistency)	AED (Diversity)	Distance
OpLGAN	0.036	0.597	−0.062	0.022
ProGen	0.048	0.394	−0.156	0.037
CVAE	0.232	0.301	0.247	0.145
ProteoGAN	0.043	0.554	−0.010	0.012
Our model	**0.034**	**0.603**	**0.006**	**0.001**

For AED and distance, we report the AED across feature dimensions and the average pairwise RKHS distance between sequences, respectively. According to these metrics, our analysis demonstrates that our model surpasses existing models in terms of similarity, consistency, and diversity. Notably, the AED results indicate that the generated sequences exhibit diversity levels comparable with, and slightly higher than, those of the real sequences. In contrast, other methods either produce sequences with significantly lower diversity (OpLGAN, ProGen, ProteoGan) or excessively high diversity (CAVE), which may indicate inferior performance in terms of similarity and conditional consistency.

We compare our model against a one-stage baseline that maps directly from function to sequence. The training procedure is illustrated in Figure S10 in the supplement. Our model exhibits faster convergence and achieves superior performance across evaluation metrics.

## Discussion

**Figure 7:**
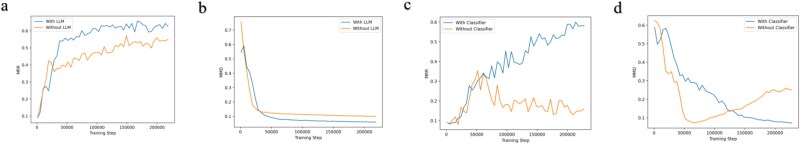
The influence of latent representation $r$ from LLM and hyperparameter $\beta $ on MRR and MMD. a. The MRR with and without LLM versus training steps; b. The MMD with and without LLM versus training steps; c. The MRR with and without classifier versus training steps; d. The MMD with and without classifier versus training steps.

We first examine the influence of the protein LLM on the outcomes of our model. For a detailed account of the results, please refer to Fig. 7. In Fig. 7a, we present the MRR values as a function of the training steps. Similarly, Fig. 7b illustrates the MMD values over the course of training. From these figures, we observe that the model’s performance improves with a representation from the protein LLM. Additionally, when comparing performance based on label conditions instead of a representation, we observe a faster convergence rate but reduced quality. We also examine the influence of different dimensions of representation in the supplementary. However, due to constraints in computational resources, we were unable to test the model with significantly higher dimensions of latent representation. We hypothesize that there may exist a threshold dimension beyond which the performance gains may plateau or diminish. Further investigation into this aspect could provide valuable insights into the optimal dimensionality for latent representations in our model.

In our analysis, we also explore the necessity of incorporating a discriminative classifier into our model. To do this, we experiment with different orders of magnitude for the hyperparameter $\beta $. Our findings indicate that increasing $\beta $ 10-fold enhances the performance of our model, suggesting that the discriminative classifier plays a crucial role in improving model efficacy. However, we observe a decline in performance when $\beta $ exceeds 300, identifying this as a threshold beyond which the benefits of increasing $\beta $ diminish.

To optimize the value of $\beta $, we employ an optimization package [34], ultimately determining that a $\beta $ value of 175 yields the best performance. This optimization process and the comparative analysis of different $\beta $ values are visually represented in Fig. 7c and d. In this figure, the x-axis represents the training steps, while the y-axis denotes the metrics, including MMD and MRR, as previously mentioned. Specifically, Fig. 7a illustrates the variation of the MRR value across training steps, and Fig. 7b displays the changes in the MMD value over the course of training.

In our study, we also investigate the potential benefits of fine-tuning ESM-2. Based on our metrics, we observe that fine-tuning ESM-2 does not significantly affect the quality of the generated sequences. This could be attributed to the fact that ESM-2 has been pretrained on millions of protein sequences, and it is likely that our training dataset might overlap with the pretrained dataset. Consequently, the representations derived from the pretrained model might already encapsulate functional information about the protein sequences, potentially offering a broader perspective than that provided by GO annotations alone. However, our investigation does not delve into optimizing the fine-tuning process for the pretrained model. There exists a possibility that enhancing the fine-tuning methodology for ESM-2 could yield different outcomes. Therefore, future research might benefit from exploring more sophisticated approaches to fine-tuning ESM-2, which could potentially lead to improved performance and more insightful conclusions.

## Conclusion

In this work, we have proposed ProteinRG, a novel hierarchical model that synergistically integrates a pretrained LLM with a generative model to facilitate the design of functional proteins when data are scarce. Our model is structured into three distinct modules: (a) the latent feature capture module, (b) the first-stage generative module, and (c) the second-stage generative module. Initially, the model generates a latent representation of a protein sequence, which is subsequently used to produce a protein sequence endowed with specific functions.

We have applied ProteinRG to a variety of functional protein sequences and evaluated the generated outcomes from three different perspectives: MSA, t-SNE distribution analysis, and 3D structures. The findings indicate that, even with a limited amount of data, ProteinRG can simultaneously ensure similarity and condition consistency in the generated protein sequences, demonstrating superior performance in comparison with other generative models for protein sequence design. Additionally, the fundamental concept of employing a large model for learning data representation, dimension reduction, and hierarchical generative modeling is broadly applicable and can be extended to other challenges, such as modeling RNA data or any type of sequence data.

Despite these promising results, our approach has certain limitations. One limitation arises when generating diverse protein families concurrently; the GO annotations database must contain a substantial number of labels to facilitate effective embedding and classification. This requirement can pose challenges related to data completeness and may incur additional costs due to the increased dimensionality of the input and output for classification. Additionally, there may be a need to refine the method of fine-tuning the ESM-2 model during the latent feature extraction stage to further enhance performance. In specific applications, such as studies targeting certain drugs through protein sequence generation, laboratory experiments are necessary to validate the generated results. While this work focuses on protein sequence data, our proposed generative model is also applicable to other sequence data, such as mRNAs. We plan to explore these aspects in future work.

**Key Points**

- The scarcity of functional protein sequence data limits the effectiveness of deep generative models for protein sequence design, as these models typically require large datasets for training.
- ProteinRG is a hierarchical model that leverages existing large protein sequence models to generate functional protein sequences even from relatively small datasets.
- Numerical experiments demonstrate that ProteinRG produces sequences similar to the originals and consistent with desired functions and diversity, outperforming other generative models.

## Supplementary Material

bib25-1008_supplement_bbag095

## Data Availability

The data that support the findings of this study are available at https://github.com/ChenyuzZZ73/ProteinRG/data. These data were derived from the following resource available in the public domain: https://www.uniprot.org/
