# Supplementary material for: De novo functional protein sequence generation: overcoming data scarcity through regeneration and large language models
Source: Brief Bioinform. 2026 Mar 8;27(2):bbag095. doi: 10.1093/bib/bbag095 (PMC12967336; doi:10.1093/bib/bbag095)
Supplement: bib25-1008_supplement_bbag095 [file bib25-1008_supplement_bbag095.pdf]

Supplementary Information for “De Novo Functional Protein Sequence Generation: Overcoming Data Scarcity through Regeneration and Large Language Models"

1. ADDITIONAL BACKGROUND INFORMATION

A. Transformers

We used a decoder-only transformer architecture in module 2 of our method. Transformers are a type of neural network architecture introduced in [1]. They were designed to enhance model performance and efficiency by enabling the focus on specific parts of the input sequence when generating each part of the output sequence. Typically, transformers have an encoder-decoder structure: the encoder processes input data to extract a representation, while the decoder generates output data based on this representation.

Self-Attention Mechanism is the key to transformers that allows models to focus on specific parts of the input sequence when generating each part of the output sequence. The self-attention mechanism is central to transformers, enabling models to focus on specific parts of the input sequence when generating each part of the output sequence. The calculation involves the following processes:

- Each input token is mapped to three vectors: Query (Q), Key (K), and Value (V).
- Calculate the attention scores using the dot product of the Query and Key vectors.
- Scale the attention scores by the square root of the dimension of the Key vectors (d_k).
- Apply softmax to obtain the attention weights.
- Multiply the attention weights by the Value vectors to obtain the final output.

The process can be formulated as:

$$\text{Attention Output} = \text{softmax}\left(\frac{QK^T}{\sqrt{d_k}}\right) \times V \tag{S1}$$

where Q, K, and V are three learnable weights of each input item. d_k is the scaled vector, which typically equals 512. Softmax is a normalization layer for attention scores.

A.1. Decoder-only transformer

The decoder-only transformer is an auto-regressive transformer model mainly used in natural language processing to deal with the task of sequence generation. One single decoder consists of a masked multi-head self-attention layer, a feed-forward layer, and a fully connected layer. In this decoder-only transformer, the input E here is a sequence that consists of the conditional function y, a time step embedding for diffusion t, a noised latent embedding vector, and a final vector as the final output for latent representation in order. The core of this network is the masked multi-head self-attention layer. The attention layer can be formulated as follows:

$$\text{head}(\mathbf{E})_i = \mathbf{W}_v^i\mathbf{E} \cdot \text{Softmax}\left[\frac{\left(\mathbf{W}_k^i\mathbf{E}\right)^\top\left(\mathbf{W}_q^i\mathbf{E}\right)}{\sqrt{\frac{d}{h}}}\right] \in R^{\frac{d}{h}\times n} \tag{S2}$$

$$\text{MultiHead}(\mathbf{E}) = \text{Concat}\ [\text{head}(\mathbf{E})_i, \ldots,\ \text{head}\ (\mathbf{E})_h] \in R^{d\times n}, \tag{S3}$$

where $\mathbf{E}$ is the input sequence mentioned above, d is the model dimension, h is the number of heads, n is the length of the sequence. $\mathbf{W}_q \in R^{d_q \times d}, \mathbf{W}_k \in R^{d_k \times d}, \mathbf{W}_v \in R^{d_v \times d}$ is the parameter matrix of attention network where $d_k = d_v = d_{\text{model}} / h$.
Then the multi-head self-attention layer is followed by a residual layer with layer normalization

$$\mathbf{Z} = \mathbf{LN}\left(\mathbf{E} + \mathbf{W}_o \cdot \text{MultiHead}\left(\mathbf{E}\right)\right) \tag{S4}$$

where $\mathbf{W}_o \in R^{d \times d}$ is a parameter of residual network. $\mathbf{Z}$ is the output of the masked multi-head self-attention layer. With a fully connected layer, the final output is the predicted representation in each time step.

B. Large language models

Recent advancements have demonstrated that LLMs develop emergent capabilities as they scale, transcending mere pattern recognition to facilitate higher-level reasoning and the generation of realistic images, text, and various sequential data forms. These models epitomize the concept of representation learning, where a task-agnostic latent representation is learned through unsupervised or self-supervised training on extensive collections of unlabeled data. Subsequently, this representation can be fine-tuned with paired data for a variety of downstream tasks, showcasing the potential for application across diverse domains. Large language models (LLMs) based on transformer architecture employ an attention mechanism to discern interaction patterns among every single item within the input text or images. Large language models usually contain billions of parameters and training data and demonstrate more powerful understanding capabilities than traditional language models, which is called a breakthrough in physics. A number of successful large language models are widely used such as ChatGPT ([2]) and LLaMA ([3]). The application of large language models (LLMs) to protein sequences has gained considerable attention in recent years. AlphaFold ([4], developed by DeepMind, AlphaFold uses deep learning and attention mechanisms to predict protein structures with high accuracy. ProtBert was ([5]) trained on large protein sequence databases to perform various prediction tasks, such as property predictions for proteins. ESM (Evolutionary Scale Modeling)([6]), created by Facebook AI Research, leverages transformer architectures to capture evolutionary patterns in protein sequences. In this work, we fine-tune the ESM-2 model using training data. The downstream task is to classify protein functions. The model receives a set of protein sequences as input and produces multi-class GO annotations as output. Specifically, we design a network architecture that includes a residual block and an MLP block with fully connected layers. The residual block consists of a linear layer with a ReLU activation and a dropout layer, which processes the output from the ESM-2 model before passing it to the fully connected classification head. The detailed network is shown in Fig.S1.

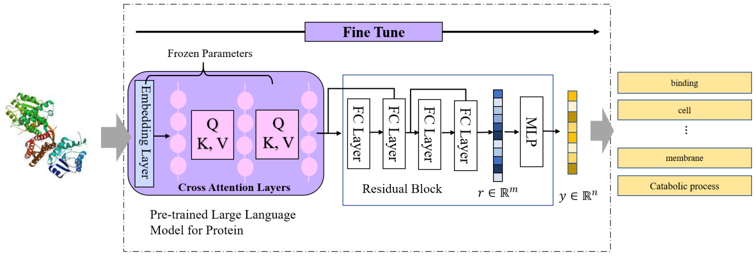

Fig. S1. Overview of Module 1: the detailed overview of the latent feature capture module.

C. Diffusion Model

We used a diffusion model to construct a representation of protein sequence data (Fig. S2). This model is a parameterized Markov chain trained through variational inference to generate samples that align with the data after a finite period. It has the form $p_\theta := \int p_\theta(x_{0:T})dx_{1:T}$ ([7]). Here

$x_1, ..., x_T$ are latents of the same dimensionality as the data $x_0 \sim q(x_0)$. The joint distribution $p_\theta(x_{0:T})$ is called the reverse process and is defined as a Markov chain with learned Gaussian transitions starting at $p(x_T) = \mathcal{N}(x_T; 0, I)$:

$$p_\theta(\mathbf{x}_{0:T}) := p(\mathbf{x}_T) \prod_{t=1}^{T} p_\theta(\mathbf{x}_{t-1} \mid \mathbf{x}_t), \quad p_\theta(\mathbf{x}_{t-1} \mid \mathbf{x}_t) := \mathcal{N}(\mathbf{x}_{t-1}; \boldsymbol{\mu}_\theta(\mathbf{x}_t, t), \Sigma_\theta(\mathbf{x}_t, t))$$

What distinguishes diffusion models from other types of latent variable models is that the approximate posterior $q(\mathbf{x}_{1:T} \mid \mathbf{x}_0)$, called the forward process or diffusion process, is fixed to a Markov chain that gradually adds Gaussian noise to the data according to a variance schedule $\beta_1, \ldots, \beta_T$:

$$q(\mathbf{x}_{1:T} \mid \mathbf{x}_0) := \prod_{t=1}^{T} q(\mathbf{x}_t \mid \mathbf{x}_{t-1}), \quad q(\mathbf{x}_t \mid \mathbf{x}_{t-1}) := \mathcal{N}\left(\mathbf{x}_t; \sqrt{1-\beta_t}\mathbf{x}_{t-1}, \beta_t \mathbf{I}\right)$$

Traditional diffusion models contain the above two steps: the forward process for training and the reverse process for sampling. While training, the key to diffusion models is to learn the real Gaussian noise added in every step t. Simply, the loss can be formulated as a mean square loss between real noise and predicted noise:

$$L(\theta) := E_{t, \mathbf{x}_0, \boldsymbol{\epsilon}}\left[\left\|\boldsymbol{\epsilon} - \boldsymbol{\epsilon}_\theta\left(\sqrt{\bar{\alpha}_t}\mathbf{x}_0 + \sqrt{1-\bar{\alpha}_t}\boldsymbol{\epsilon}, t\right)\right\|^2\right]$$

where t is uniform between 1 and T and ϵ is sampled from $\mathcal{N}(0, I)$. Some applications based on the traditional diffusion model have shown promising improvement in image generation and text-to-image generation ([8–10]). In our specific application of the diffusion model, we have found it more effective to predict the final latent representation r directly, rather than predicting the noise in the forward process.

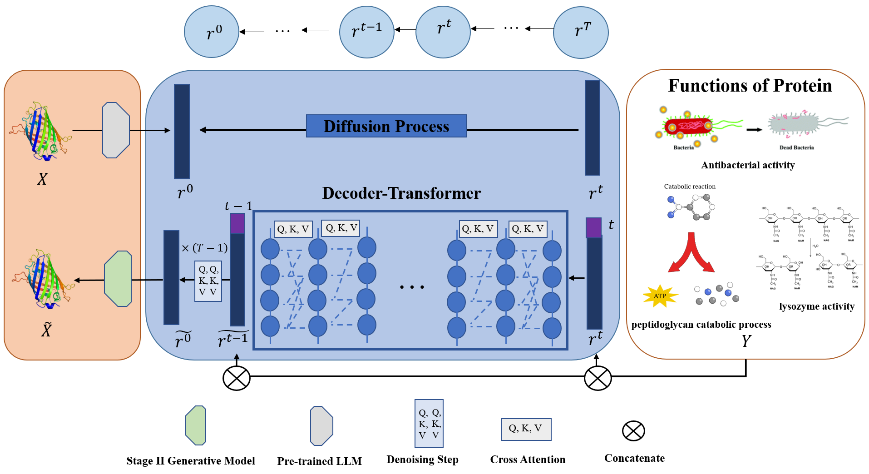

Fig. S2. Overview of Module 2: learning protein sequence representations given annotations.

D. CWGAN-GP

CWGANs are composed of two neural networks: a generator and a discriminator. The training of CWGAN-GP involves a minimax game where the generator seeks to minimize the Wasserstein distance with a gradient penalty between the real and generated data distributions, while the discriminator aims to maximize this distance.

In the CWGAN-GP, both the generator and the discriminator receive a condition as part of their input. In this work, the condition is specified as the latent representation R and annotation Y of a protein sequence. To simplify the notation, let $U = (R, Y)$. The whole loss function can be written as follows:

$$V(G, D) = \underset{(Z,U)\sim P_Z P_U}{E}[D(G(Z,U),U] - \underset{(X,U)\sim P_{(X,U)}}{E}[D(X,U)] + \lambda \text{Pen}(D), \quad \text{(S5)}$$

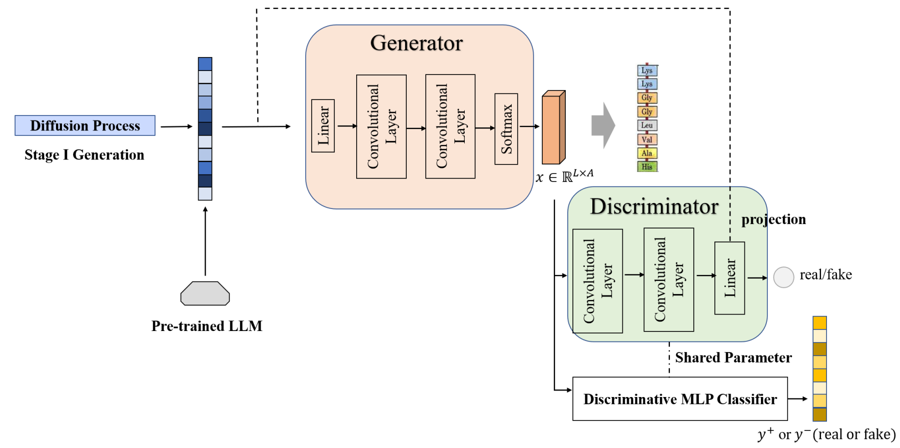

Fig. S3. Overview of Module 3: a conditional Wasserstein Generative Adversarial Network with an auxiliary multi-class discriminative classifier is trained to generate protein sequences given the annotation.

where $Z \sim P_Z = N(\mathbf{0}, \mathbf{I})$ is a Gaussian noise vector, $\lambda \geq 0$ is a tuning parameter, and

$$\text{Pen}(D) = \underset{(\hat{X},Y)\sim P_{\hat{X}}P(Y)}{E}\left[\left(\left\|\nabla_{\hat{X}} D_\eta(\hat{X}, Y)\right\|_2 - 1\right)^2\right]$$

is a penalty term to ensure the Lipschitz regularity of the discriminator function D.

To enhance conditional consistency and address the issue of low intra-class diversity, we propose the integration of an auxiliary discriminative classifier capable of handling multiple classes, see Fig. S3.

The objective functions for the generator, the discriminator, and the discriminative classifier are defined as follows:

$$\max_{D, C_\mathrm{d}} \left\{ V(G, D) + \beta \cdot \{ E_{(X,Y)\sim P_{X,Y}}[\log C_\mathrm{d}(Y^+ \mid X)] + E_{(X,Y)\sim P_{X,Y}}[\log C_\mathrm{d}(Y^- \mid X)] \} \right\},$$

$$\min_{G} \left\{ V(G, D) - \beta \cdot \{ E_{(X,Y)\sim P_{X,Y}}[\log C_\mathrm{d}(Y^+ \mid X)] - E_{(X,Y)\sim P_{X,Y}}[\log C_\mathrm{d}(Y^- \mid X)] \} \right\},$$

where $V(G, D)$ represents the CWGAN-GP objective function defined in the Supplementary Materials, Y^+ is a binary label indicating the real protein sequence or the generated one, Y^- is a one-hot vector representing the types of the generated multi-class protein sequences, and a hyperparameter β is introduced to balance the influence of the discriminator and the classifier.

2. DETAILED DATASETS

A. Lysozyme sequences

The lysozyme sequence dataset from the UniProtKB database consists of two most prevalent types: Lysozyme C and Lysozyme G. Within this database, we identified 1097 annotated sequences for Lysozyme C and 1668 for Lysozyme G. We excluded sequences containing non-standard amino acids (U, J, Z, O, B, X) to ensure data quality. Consequently, our training dataset consists of 2249 lysozyme sequences. Additionally, we set aside 516 lysozyme sequences for validation purposes, maintaining the same class distribution as observed in the original dataset. For our dataset, we identified 60 GO annotations related to lysozyme sequences, encompassing functions such as lysozyme activity (GO:0003796), defense response to bacterium (GO:0042742), killing of cells of another organism (GO:0031640), metabolic process (GO:0008152), digestion (GO:0007586), among others. Fig. S4 presents the detailed GO functions for both types of lysozyme sequences. From this figure, it is evident that both Lysozyme C and Lysozyme G share the function of lysozyme activity (GO:0003796). However, distinct functions are also observed; for instance, Lysozyme G is predominantly associated with the peptidoglycan catabolic process (GO:0009253), which involves the breakdown of peptidoglycans.

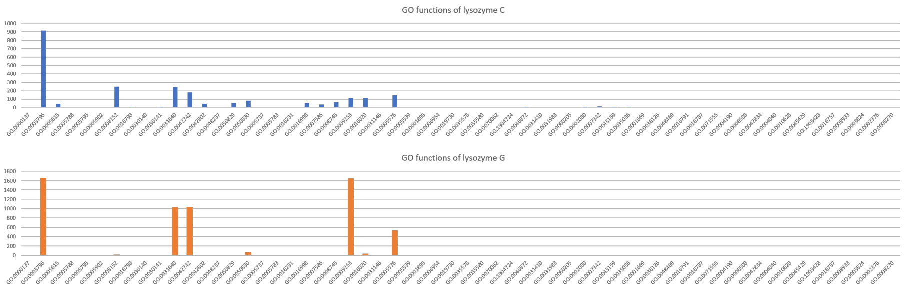

Fig. S4. GO annotations of lysozyme C and lysozyme G. The upper panel is for lysozyme C and the lower one is for lysozyme G.

B. Malate dehydrogenase (MDH) sequences

MDH is a crucial enzyme in cellular metabolism that catalyzes the reversible oxidation of malate to oxaloacetate. Sequences are downloaded from UniProtKB with 15,000 MDH sequences for training and 1,722 for validation. Among all these sequences, we include 11 GO annotations related to MDH as input labels, which include Malate metabolic process (GO:0006108), NADH metabolic process (GO:0006734), L-malate dehydrogenase activity (GO:0030060), Malate dehydrogenase activity (GO:0046554) and so on. The detailed data can be found in the supplementary data files.

For our dataset, we identified 60 GO annotations related to lysozyme sequences, encompassing functions such as lysozyme activity (GO:0003796), defense response to bacterium (GO:0042742), killing of cells of another organism (GO:0031640), metabolic process (GO:0008152), digestion (GO:0007586), among others. Figure S4 presents the detailed GO functions for both types of lysozyme sequences. From this figure, it is evident that both Lysozyme C and Lysozyme G share the function of lysozyme activity (GO:0003796). However, distinct functions are also observed; for instance, Lysozyme G is predominantly associated with the peptidoglycan catabolic process (GO:0009253), which involves the breakdown of peptidoglycans.

3. TRAINING

Our network training is conducted on four Nvidia GeForce A100 GPUs. The training process is divided into two stages, with the first stage module and the second stage being trained independently. We utilize the pre-trained large language model ESM-2 (esm2_t12_35M_UR50D), which is equipped with 12 self-attention layers and encompasses 35 million parameters. The output generated by this pre-trained large language model is a one-dimensional continuous vector. Throughout the fine-tuning process, we keep the ESM-2 weights fixed, with the exception of those in the final layers.

To enhance the training of the first stage module, we augment the representation to 512 dimensions by appending zeros. Consequently, the output from the latent feature capture module is transformed into a 512-dimensional vector. This adjustment ensures that the initial stage of the network is better equipped to process and learn from the input data, facilitating more effective training and potentially leading to improved performance in subsequent tasks.

In our experiments, we found that batch sizes of both 64 and 128 worked best for the generation tasks. Also, among various learning rates, 1e-5 seemed to be the best fit. The different values are summarized in the TableS1 above.

4. EVALUATION METRICS

We employed various metrics to evaluate the quality of the generated protein sequence data. Detailed descriptions of these metrics are provided below.

Evaluating distribution similarity and conditional consistency

Since the primary objective of generative models is to accurately replicate the distribution of target data, it is logical to evaluate their performance using a statistical two-sample test that compares the distributions of both the generated data and the training data. For protein sequence data, which poses challenges for direct analysis, this evaluation can be effectively conducted on extracted feature vectors. The Maximum Mean Discrepancy (MMD) [11] serves as a test statistic

Hyperparameter	Values
Learning Rate	0.001, 0.0004, 0.0001, 0.00004, 0.00001
Batch Size	32, 64, 128
Latent Dimension	10, 320, 640
β	1, 10, 100, 135, 175, 200

Table S1. Different Values of the hyperparameters for the model

for this purpose, comparing the mean embeddings within a Reproducing Kernel Hilbert Space (RKHS).

In the context of conditional generation, it is crucial to evaluate the model's ability to produce sequences that align with specific target labels. To this end, the Mean Reciprocal Rank (MRR) [12] enhances the Maximum Mean Discrepancy (MMD) metric by calculating the MMD between subsets of sequences corresponding to each label. This involves ranking the Reproducing Kernel Hilbert Space (RKHS) distance between generated samples and their designated target label in comparison to distances from off-target labels. Essentially, this metric evaluates how frequently sets of real sequences with off-target labels are distributionally closer to the generated sequences than the real sequences bearing the target label.

.1. The Maximum Mean Discrepancy (MMD)

Consider $R = \{r_i\}_{i=1}^{n}$ and $G = \left\{g_j\right\}_{j=1}^{m}$ as samples from the distributions of real and generated protein sequences, denoted by P_r and P_g, respectively. The MMD statistic is then defined as:

$$\mathrm{MMD}^2(R, G) = \left\| \frac{1}{n} \sum_{i=1}^{n} \phi\left(r_i\right) - \frac{1}{m} \sum_{j=1}^{m} \phi\left(g_j\right) \right\|_2^2. \tag{S6}$$

This equation quantifies the squared distance between the mean embeddings of real and generated protein sequences in the RKHS, providing a measure of the discrepancy between the two distributions. Since the length of protein sequence data could reach 2000, we apply the Spectrum kernel to represent the extracted feature vectors for faster computing compared with other embedding methods.

.2. The Mean Reciprocal Rank (MRR)

The Mean Reciprocal Rank (MRR) extends the MMD metric by computing MMD between subsets of sequences for each label and ranking the RKHS distance between generated samples and their target label among distances to off-target labels. It measures how many sets of real sequences with off-target labels are closer in distribution to the generated sequences than real sequences with the target label.

Let R represent a set of real sequences Ri, each annotated with a label $i \in 1, \ldots, d$, where d denotes the total number of labels. Let $G = \{G_i\}_{i=1}^{d}$ be a correspondingly structured set of generated sequences. Our objective is to maximize the Mean Reciprocal Rank (MRR) as defined by:

$$\mathrm{MRR}(R, G) = \frac{1}{d} \sum_{i=1}^{d} \frac{1}{\mathrm{rank}_R\left(\mathrm{MMD}\left(R_i, G_i\right)\right)}, \tag{S7}$$

where $\mathrm{rank}_R(\mathrm{MMD}(R_i, G_i))$ represents the rank of $\mathrm{MMD}\left(R_i, G_i\right)$ within the sorted list $\{\mathrm{MMD}(R_1, G_i), \ldots, \mathrm{MMD}(R_d, G_i)\}$. The MRR value reaches its maximum of 1 when the distributions of generated protein sequences for a given label are closest to the distribution of real protein sequences with the same label, indicating optimal alignment between generated sequences and their intended labels.

A. Shannon's entropy

The formula for calculating Shannon's entropy is as follows:

$$H = -\sum(P(i) \cdot \log_2(P(i))), \tag{S8}$$

where H represents the entropy value at a specific position in the alignment. The probability of observing a particular residue i at a given position is expressed as $P(i)$. The entropy calculation involves summing over all possible residues that can occur at that position.

B. Multiple Sequence Alignment Entropy

Multiple Sequence Alignment (MSA) entropy is a quantitative measure designed to assess the informational content embedded within a multiple sequence alignment. The computation of MSA entropy uses Shannon's entropy formula, a method that calculates the average informational content necessary to describe the state of a random variable. In the context of MSA, this random variable pertains to the residues occupying a particular position in the alignment. This approach allows for a systematic evaluation of the conservation and diversity present within a multiple sequence alignment, providing critical insights into the structural and functional aspects of the sequences involved.

C. TM-Score

TM-score is a length-normalized measure of global structural similarity between two protein conformations. After optimal rigid-body superposition, it is defined as

$$\text{TM-score} = \max_{\text{superpositions}} \left\{ \frac{1}{L_{\text{target}}} \sum_{i=1}^{L_{\text{aligned}}} \frac{1}{1 + \left(\frac{d_i}{d_0(L_{\text{target}})} \right)^2} \right\},$$

where d_i is the Euclidean distance between matched Cα atoms, L_{target} is typically the target length, and the length-dependent scale is

$$d_0(L) = 1.24\,(L - 15)^{1/3} - 1.8.$$

Compared with RMSD, TM-score employs a saturating distance penalty, reducing the impact of outliers and mitigating length dependence, thereby better capturing overall fold topology. It is widely used for model evaluation, template search, and structural clustering. Common thresholds are:

- TM-score ≥ 0.5: likely the same overall fold/topology;
- $0.3 \leq$ TM-score < 0.5: partial global similarity;
- TM-score < 0.3: close to random alignment.

IDDT

The IDDT (Identification of Directly Determined Contacts) [13] metric is commonly employed to evaluate the concordance between contact predictions made by AlphaFold and those determined through experimental methods. The analysis is initiated by compiling a dataset that includes protein structures with experimentally determined contacts. Subsequently, the IDDT score is computed by measuring the degree of overlap between the contacts predicted by AlphaFold and the experimentally observed contacts. Higher IDDT scores signify a stronger correlation between the predicted and experimental contacts, which implies a higher level of accuracy in the predicted protein structure. By examining the distribution, mean values, and trends of IDDT scores across the dataset, researchers can derive valuable insights into the general predictive performance of AlphaFold in the realm of protein structure determination.

Delta Delta G (DDG)

Delta Delta G (DDG) quantifies how a single amino-acid substitution perturbs protein thermodynamic stability. Formally, it is defined as the difference in folding free energy between the mutant and wild-type proteins,

$$DDG = DG_{fold}(mutant) - DG_{fold}(wildtype) \tag{S9}$$

where

$$DG_{fold} = G_{folded} - G_{unfolded} \tag{S10}$$

Thus, DDG captures a "change in a change": the first change reflects the energetic cost of folding, and the second reflects how that cost shifts upon mutation. By convention, positive DDG values indicate destabilization (the mutant folds less favorably), whereas negative values indicate stabilization. Reported in kcal/mol under specified solution conditions, DDG has emerged as a practical surrogate for predicting whether a point mutation will be favorable for stability, and it correlates though not perfectly with experimental readouts such as melting temperature (Tm) and chemical denaturation midpoints. When interpreting or benchmarking predictions, it is important to consider method-specific sign conventions, the uncertainty of the estimate (e.g., standard error across replicates), and performance metrics such as Pearson/Spearman correlation with experimental data and mean absolute error. Structural context also matters: predictions are more reliable in well-resolved or high-confidence regions and may degrade near flexible termini, ligand-binding sites, or oligomeric interfaces not explicitly modeled. Consequently, robust stability design workflows often combine multiple predictors, emphasize consistency in the direction of effect, and validate top-ranked mutations experimentally.

5. ADDITIONAL RESULTS

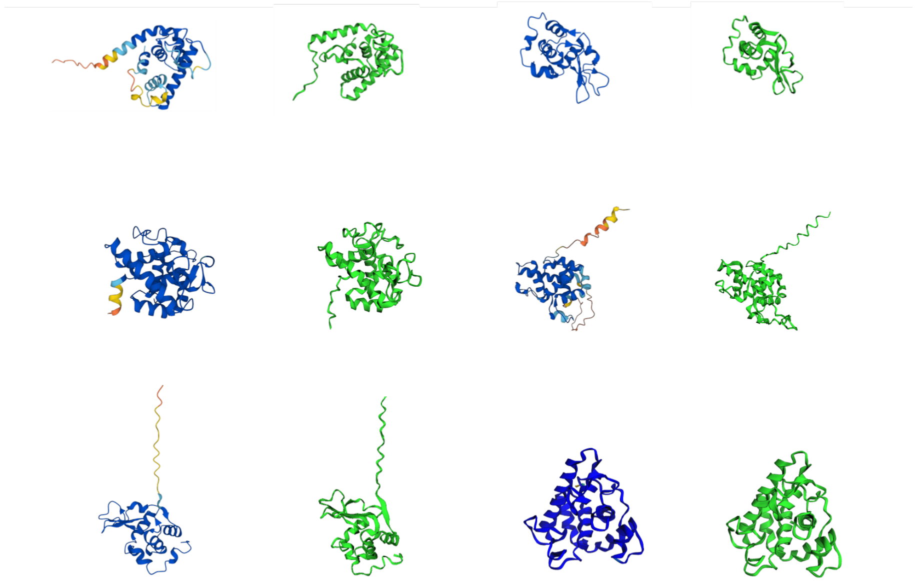

Fig. S5. The 3D structure prediction results of the natural lysozyme sequences and the generated lysozyme sequences from ProteinRG. The blue one is the natural one on the left, and the green one is the generated one on the right. The identity of generated sequences can reach 90%.

We provide additional results showcasing the 3D structures of several generated sequences, along with comparisons to natural sequences sharing the same GO annotations. The prediction results for the 3D structures of these sequences are analyzed in Fig.S5. For Figures S5, the results for the real lysozyme sequences are displayed on the left, while those for the generated sequences are on the right. The detailed 3D structure predictions by AlphaFold2 are further showcased in Figure S5b, where a visual comparison reveals a high degree of similarity between the generated and natural protein structures, where the identity of those generated lysozyme sequences can reach 90%.

We further analyse the similarity between the natural one and the generated one with structure alignment. We calculate the TM-score of four groups of natural protein and generated one using TM-alignment [14]. The results of TM-score are shown in Fig. S6. The gray one means a disalignment between two proteins, while other colors mean different kinds of secondary structure. TM-score infers that the structures between natural protein and the generated one are consistent in the majority part, which guarantees the functional motifs and bioactivity. Besides, we compared the stability of generated and natural sequences, explicitly incorporating prediction confidence as a quality-control measure in Figure S7a. We plotted violin charts and ran statistical

tests for both datasets and a high-confidence subset (e.g., Confidence ≥ 0.85). The two groups show largely overlapping DDG distributions with small differences in mean and median. In lysozyme, the Generated group is slightly left-shifted (lower DDG), while in MDH the two groups are broadly comparable with a similar spread. These patterns indicate that the generated sequences are, on average, as stable as their natural counterparts. The confidence distributions are similar between groups, indicating that the results are not driven by low-confidence predictions.

Regarding the novelty of generated protein sequences, We quantify local novelty using a sliding-window percent identity to the nearest training sequence in Fig. S7b. This captures how similar each segment of the query is to known sequences. The heatmap encodes percent identity along the sequence (x-axis). Warmer colors indicate higher local identity; cooler colors indicate divergence. The alternating bands reveal segments that closely match known sequences versus segments where the model introduces variation. Together with Shannon entropy, positions with high identity and low entropy mark tightly constrained sites where conservation is expected. Positions with lower identity and higher entropy mark flexible regions where diversity is tolerated and generated.

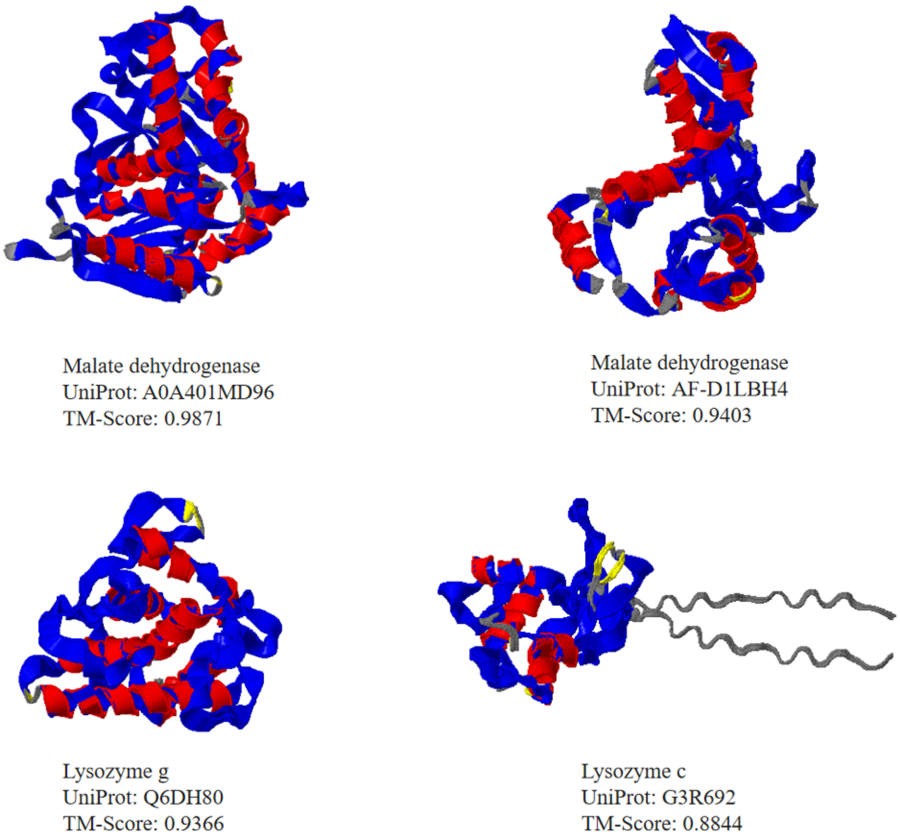

Fig. S6. The alignment results of the natural protein structure and the generated protein structure. The gray one means misalignment between two proteins, while other colors mean different kinds of secondary structure.

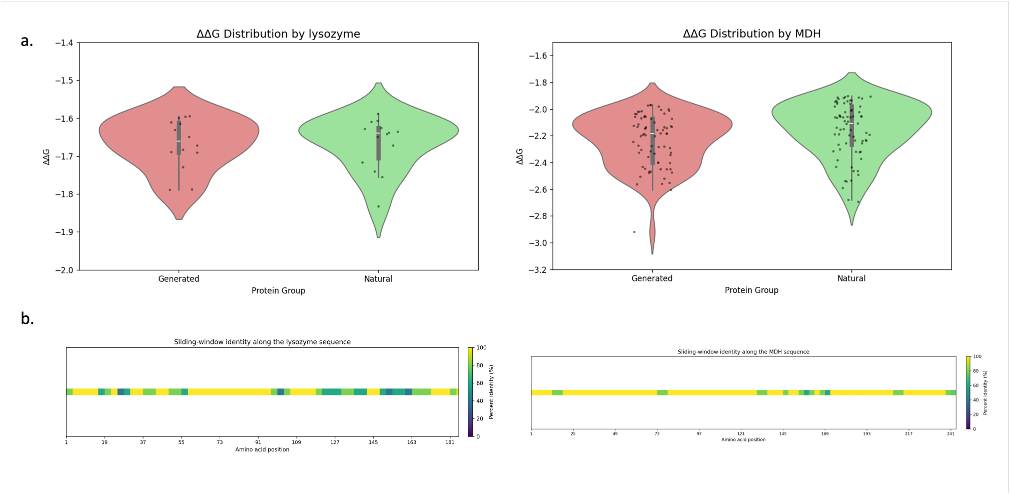

Fig. S7. a. Violin plots compare the distribution of DDG values for Generated and Natural sequences in two enzyme systems (left: lysozyme; right: MDH). b. Sliding-window percent-identity tracks along the reference sequences

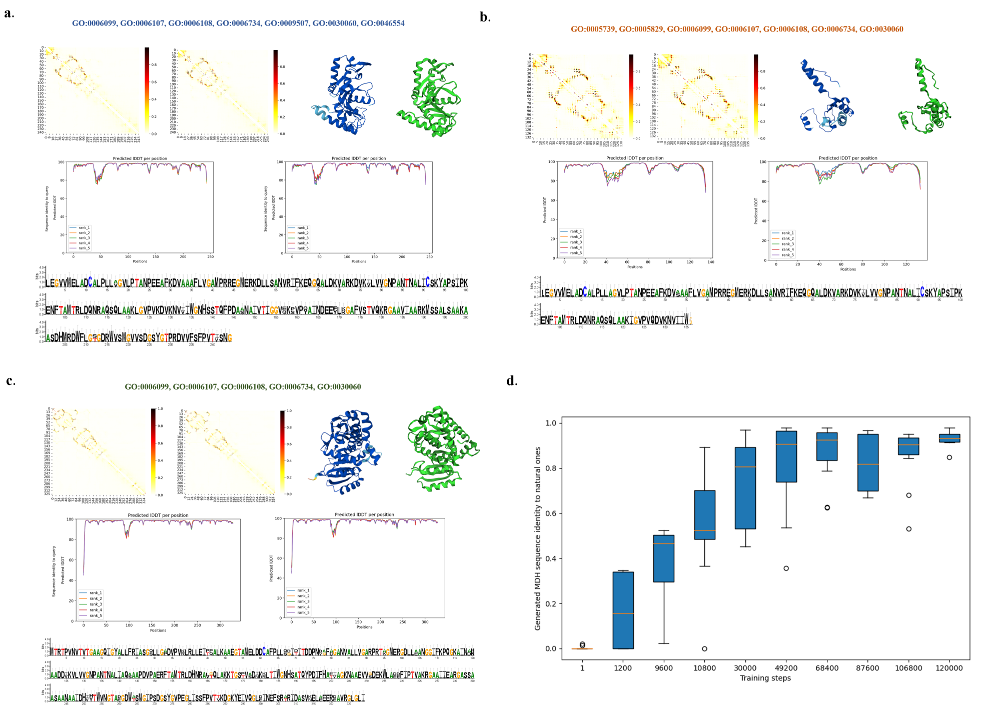

Fig. S8. The analysis results of the natural MDH sequence (left) and the generated MDH sequence (right). The attention map, IDDT at each position, conversation analysis and 3D-structure prediction from Alphafold2 are given in **a**, **b**, and **c**. **a**. The input GO annotations for the protein sequence include GO:0006099, 0006107, 0006108, 0006734, 0009507, 0030060, and 0046554. **b**. The input GO annotations include GO:0005739, 0005829, 0006099, 0006107, 0006108, 0006734, and 0030060. **c**. The input GO annotations include GO:0006099, 0006107, 0006108, 0006734, and 0030060. **d**. The similarity of the 32 generated MDH sequences to the natural ones from test data at different training steps.

Fig. S8a, S8b, and S8c present the analysis results, with the natural MDH sequence on the left

and the generated MDH sequence on the right. The attention map, showing the 2D interactions between each amino acid of the sequence, is predicted by ESM-2. The IDDT scores and 3D structure predictions are conducted by AlphaFold2. Sequence conservation analysis is based on MSA and MEME suit [15] for one natural MDH sequence and one generated MDH sequence with the same GO annotations. We performed de novo motif discovery with MEME on the natural sequences for each GO-defined group and used the resulting PWMs to scan both natural and generated sequences. Additionally, Fig. S8d presents the sequence identity of the generated MDH sequences to the natural MDH sequences from the test dataset at different training steps. The number of selected generated sequences is 32. We calculate the MDH sequence identity using the Biopython package ([16]). The final identity of the generated MDH sequences can reach up to 95%.

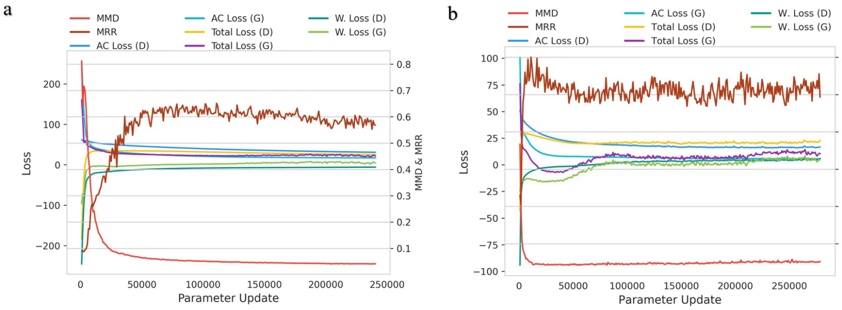

Fig. S9. The loss and the metric results of our model on the left and ProteoGAN on the right with training step

The training process is shown in Fig. S9. The x-axis is the training step. The y-axis is the performance metrics, including Maximum Mean Discrepancy (MMD), Mean Reciprocal Rank (MRR), generator loss, discriminator loss from WGAN-GP, and the cross entropy from the classifier. We also discuss the ability of our model in small datasets including lysozyme and MDH protein sequences. We compare our model with one-stage model where the input is function directly. The training process is shown in Fig. S10. We found that our model performence better in the the converge rate and evlauation metrics.

We have selected five distinct types of protein sequences from the dataset, each associated with different functions, to demonstrate the learning capabilities of our model with limited data. Each type of protein sequence is represented by approximately one thousand samples for training purposes.

Fig. S11a illustrates proteins involved in drug binding and transmembrane transporter activity. Figure S11b showcases proteins with catalytic activity, cofactor binding, and anion binding functions. Fig. S11c highlights proteins that are associated with small molecule binding, signaling receptor activity, catalytic activity with a focus on proteins and kinase activity. Fig. S11d presents proteins functioning in ion transmembrane transporter activity and cation binding. Lastly, Fig. S11e depicts proteins with DNA binding and RNA binding capabilities. Fig. S11f provides an overview of the distribution of these five types of protein sequences, offering insights into the diversity and specialization of functions within the dataset.

In our analysis, we also explore the necessity of incorporating a discriminative classifier into our model. To do this, we experiment with three different orders of magnitude for the hyperparameter β. Our findings indicate that increasing β tenfold enhances the performance of our model, suggesting that the discriminative classifier plays a crucial role in improving model efficacy. However, we observe a decline in performance when β exceeds 300, identifying this as a threshold beyond which the benefits of increasing β diminish.

To optimize the value of β, we employ an optimization package [17], ultimately determining that a β value of 175 yields the best performance. This optimization process and the comparative analysis of different β values are visually represented in Fig. S12 c and d. In this figure, the x-axis represents the training steps, while the y-axis denotes the metrics, including Maximum Mean Discrepancy (MMD) and Mean Reciprocal Rank (MRR), as previously mentioned. Specifically, Fig. S12a illustrates the variation of the MRR value across training steps, and Fig. S12b displays

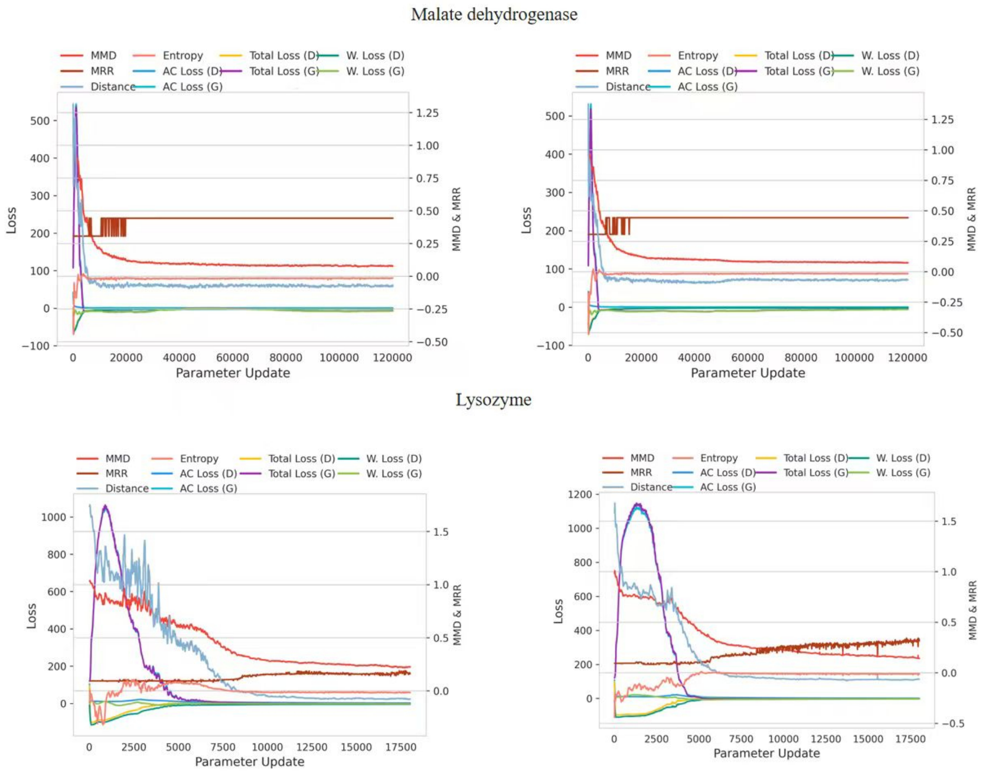

Fig. S10. The influence of one-stage model (left) and two-stage model (right) for small datasets. In the one-stage, the input of model includes the GO annotations while the input of two-stage model is latent representations of protein sequences.

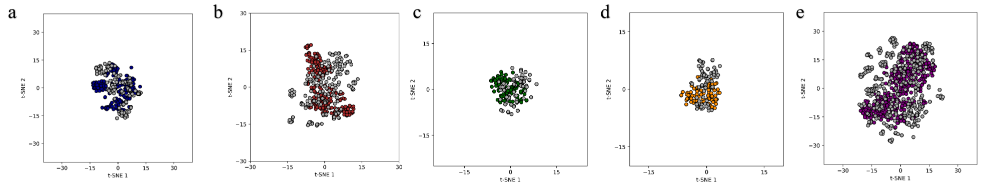

Fig. S11. t-SNE visualization from natural protein and generated protein with five different functions. a. Drug binding and transmembrane transporter activity. b. Catalytic activity, cofactor binding, and anion binding. c. Small molecule binding, signaling receptor activity, catalytic activity, acting on a protein and kinase activity. d. Ion transmembrane transporter activity and cation binding. e. DNA binding and RNA binding.

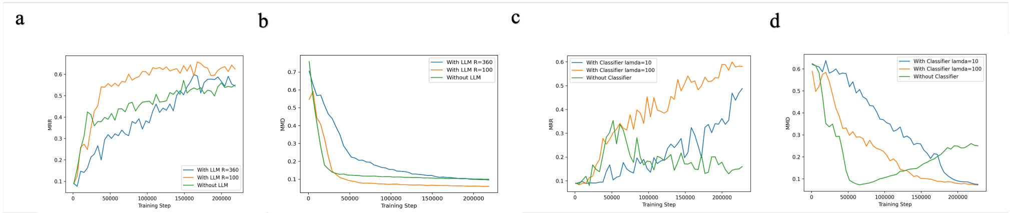

Fig. S12. The influence of the dimension of latent representation r and hyperparameter β on MRR and MMD. a. The MRR with different r values versus training steps; b. The MMD with different r values versus training steps; c. The MRR with different β values versus training steps; d. The MMD with different β values versus training steps.

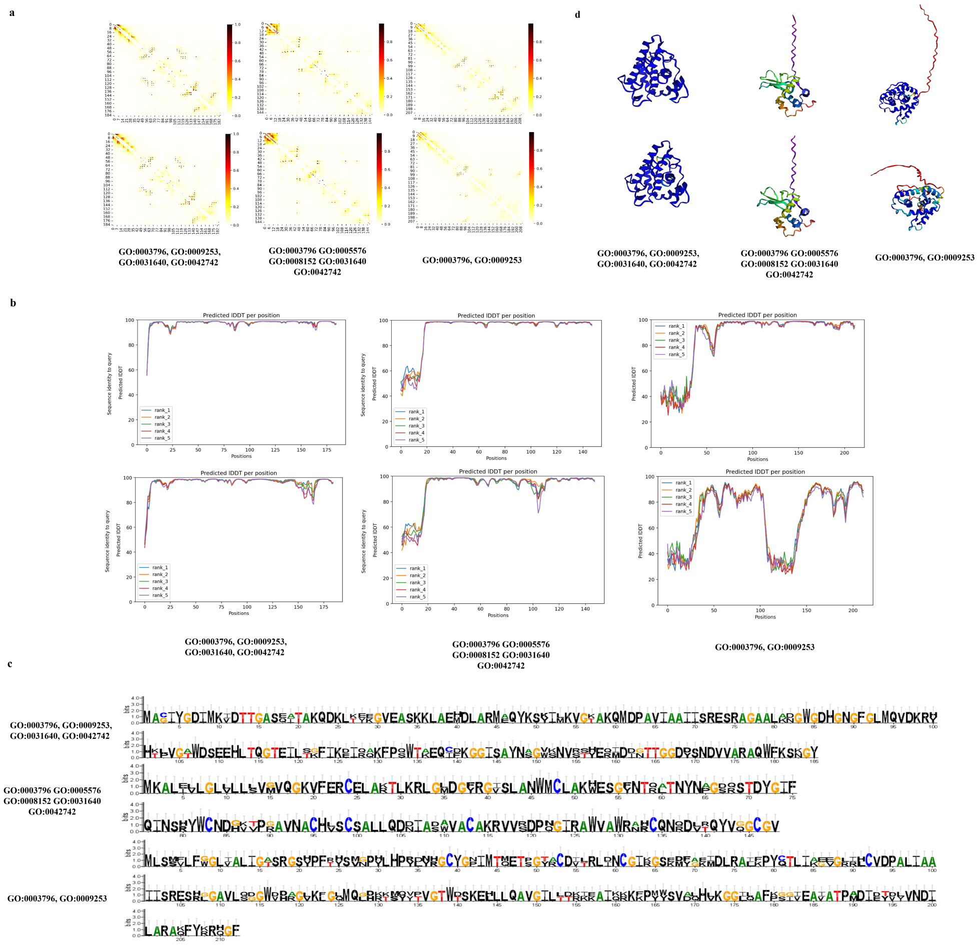

Fig. S13. a. The attention map for the natural lysozyme above and the generated lysozyme below. A larger value indicates that the amino acid residue at a position on the x-axis is in contact with the amino acid residue at a position on the y-axis. b. The IDDT value for each position: the natural lysozyme above and the generated lysozyme below. c. The results of the conservation analysis based on MSA for one natural lysozyme and one generated lysozyme, highlighting different gene ontology terms. d. Detailed 3D structure predictions by AlphaFold2, with the natural structure above and the generated prediction below.

the changes in the MMD value over the course of training.

Fig. S13 shows (a) the attention map for the natural lysozyme above and the generated lysozyme below. A larger value indicates that the amino acid residue at a position on the x-axis is in contact with the amino acid residue at a position on the y-axis. (b) The IDDT value for each position: the natural lysozyme above and the generated lysozyme below. (c) The results of the conservation analysis based on MSA for one natural lysozyme and one generated lysozyme, highlighting different gene ontology terms. (d) Detailed 3D structure predictions by AlphaFold2, with the natural structure above and the generated prediction below.

Fig. S14 shows the contact map with 3D structure input (left) and attention map with protein sequence input.

REFERENCES

1. A. Vaswani, N. Shazeer, N. Parmar, *et al.*, "Attention is all you need," in *Advances in Neural Information Processing Systems,* vol. 30 (2017).
2. OpenAI, "Anything about chatgpt," [Online]. https://help.openai.com/en/collections/3742473-chatgpt.
3. Meta, "llama models in hugging face," [Online]. https://huggingface.co/meta-llama.

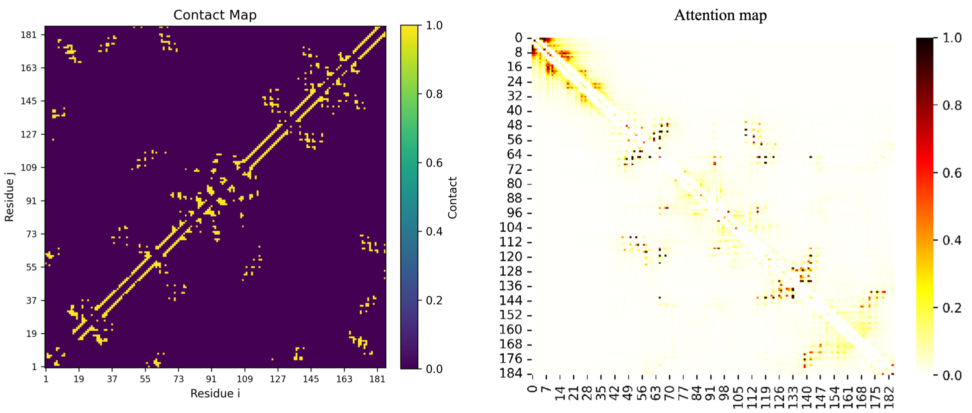

Fig. S14. Contact map with 3D structure input (left) and attention map with protein sequence input.

4. J. Jumper, R. Evans, A. Pritzel, *et al.*, "Highly accurate protein structure prediction with AlphaFold," Nature **596**, 583–589 (2021).
5. N. Brandes, D. Ofer, Y. Peleg, *et al.*, "Proteinbert: a universal deep-learning model of protein sequence and function," Bioinformatics **38**, 2102–2110 (2022).
6. Z. Lin, H. Akin, R. Rao, *et al.*, "Language models of protein sequences at the scale of evolution enable accurate structure prediction," bioRxiv (2022).
7. J. Ho, A. Jain, and P. Abbeel, "Denoising diffusion probabilistic models," in *Advances in Neural Information Processing Systems,* vol. 33 (2020), pp. 6840–6851.
8. A. Ramesh, P. Dhariwal, A. Nichol, *et al.*, "Hierarchical text-conditional image generation with CLIP latents," arXiv 2204.06125 (2022).
9. J. Ho and T. Salimans, "Classifier-free diffusion guidance," arXiv preprint arXiv:2207.12598 (2022).
10. R. Rombach, A. Blattmann, D. Lorenz, *et al.*, "High-resolution image synthesis with latent diffusion models," (2021).
11. A. Gretton, K. M. Borgwardt, M. J. Rasch, *et al.*, "A kernel two-sample test," J. Mach. Learn. Res. **13**, 723–773 (2012).
12. T. Kucera, M. Togninalli, and L. Meng-Papaxanthos, "Conditional generative modeling for de novo protein design with hierarchical functions," Bioinformatics **38**, 3454–3461 (2022).
13. M. Weigt, R. A. White, H. Szurmant, *et al.*, "Identification of direct residue contacts in protein–protein interaction by message passing," Proc. Natl. Acad. Sci. **106**, 67–72 (2009).
14. Y. Zhang and J. Skolnick, "Tm-align: a protein structure alignment algorithm based on the tm-score," Nucleic acids research **33**, 2302–2309 (2005).
15. T. L. Bailey, J. Johnson, C. E. Grant, and W. S. Noble, "The meme suite," Nucleic acids research **43**, W39–W49 (2015).
16. P. J. Cock, T. Antao, J. T. Chang, *et al.*, "Biopython: freely available python tools for computational molecular biology and bioinformatics," Bioinformatics **25**, 1422 (2009).
17. S. Falkner, A. Klein, and F. Hutter, "Bohb: Robust and efficient hyperparameter optimization at scale," in *International Conference on Machine Learning,* (PMLR, 2018), pp. 1437–1446.

- Fig S1 caption: Overview of Module 1: the detailed overview of the latent feature capture module.
  ALT Text: A schematic diagram illustrating the network architecture of the latent feature capture module. On the left, a protein structure is shown next to an embedding layer. The central block shows a pre-trained Large Language Model for Protein containing cross-attention layers with Query (Q), Key (K), and Value (V) components. To the right, the output passes through a residual block containing a ReLU layer, Dropout, and FC Layer, followed by an MLP block leading to a final classification output represented by terms like "binding," "cell," and "membrane."

- Fig. S2 caption: Overview of Module 2: learning protein sequence representations given annotations.
  ALT Text: A diagram explaining the diffusion process and decoder-transformer architecture. The top section shows a diffusion process moving from latent representation r_0 to r_T. The central block details the Decoder-Transformer, which takes inputs including time step t, noised latent embedding, and conditional functions y. It utilizes masked multi-head self-attention layers (Q, K, V) to predict the final latent representation. The bottom shows the integration of the Stage II Generative Model, Pre-trained LLM, Denoising Step, and Cross Attention to output generated code.

- Fig. S3 caption: Overview of Module 3: a conditional Wasserstein Generative Adversarial Network with an auxiliary multi-class discriminative classifier is trained to generate protein sequences given the annotation.
  ALT Text: A flowchart of the Generative Adversarial Network (GAN) architecture. The top section shows a Generator receiving input from the Diffusion Process and Stage I Generation. The Generator consists of linear and convolutional layers. The bottom section shows a Discriminator with convolutional and linear layers that output a real/fake decision (y^+ or y^-) and a Discriminative MLP Classifier that shares parameters with the discriminator to classify protein types.

- Fig. S4 caption: GO annotations of lysozyme C and lysozyme G. The upper panel is for lysozyme C and the lower one is for lysozyme G.
  ALT Text: Two bar charts displaying Gene Ontology (GO) annotations. The top chart shows blue bars representing the frequency of various GO terms for Lysozyme C. The bottom chart shows orange bars representing the frequency of GO terms for Lysozyme G. Both charts share the function of lysozyme activity (GO:0003796), while Lysozyme G shows a distinct peak for peptidoglycan catabolic process.

- Fig. S5 caption: The 3D structure prediction results of the natural lysozyme sequences and the generated lysozyme sequences from ProteinRG. The blue one is the natural one on the left, and the green one is the generated one on the right. The identity of generated sequences can reach 90
  ALT Text: A visual comparison of protein structures. The image displays several pairs of 3D protein models. In each pair, a blue ribbon structure representing the natural lysozyme sequence is positioned on the left, and a green ribbon structure representing the generated sequence is positioned on the right, showing high structural similarity between the pairs.

- Fig. S6 caption: The alignment results of the natural protein structure and the generated protein structure. The gray one means disalignment between two proteins, while other colors mean different kinds of secondary structure.
  ALT Text: Four 3D protein structure alignment visualizations. The top row shows Malate dehydrogenase comparisons with TM-Scores of 0.9871 and 0.9403. The bottom row shows Lysozyme comparisons with TM-Scores of 0.9366 and 0.8844. The structures are superimposed, with red and blue regions indicating matching secondary structures (helices and sheets) and gray regions indicating areas where the structures do not align perfectly.

- Fig. S7 caption: a. Violin plots compare the distribution of DDG values for Generated and Natural sequences in two enzyme systems (left: lysozyme; right: MDH). b. Slidingwindow percentidentity tracks along the reference sequences.
  ALT Text: Two sets of data visualizations. Panel (a) contains two violin plots showing the distribution of Delta Delta G (DDG) values; the left plot compares Generated vs. Natural for Lysozyme (red), and the right plot compares them for MDH (green). Panel (b) shows two heatmaps representing sliding-window percent identity along the protein sequences, with color gradients ranging from purple/blue (low identity) to yellow (high identity).

- Fig. S8 caption: The analysis results of the natural MDH sequence (left) and the generated MDH sequence (right). The attention map, lDDT at each position, conversation analysis and 3D-structure prediction from Alphafold2 are given in a, b, and c. a. The input GO annotations for the protein sequence include GO:0006099, 0006107, 0006108, 0006734, 0009507, 0030060, and 0046554. b. The input GO annotations include GO:0005739, 0005829, 0006099, 0006107, 0006108, 0006734, and 0030060. c. The input GO annotations include

GO:0006099, 0006107, 0006108, 0006734, and 0030060. d. The similarity of the 32 generated MDH sequences to the natural ones from test data at different training steps.
ALT Text: A multi-panel figure showing protein analysis. Panels a, b, and c each display a comparison between a natural (left) and generated (right) MDH sequence, including triangular attention maps, line graphs of lDDT scores, sequence logos, and 3D protein structures. Panel d is a box plot showing the increasing sequence identity of generated MDH sequences relative to natural ones across increasing training steps (from 1200 to 120000).

- Fig. S9 caption: The loss and the metric results of our model on the left and ProteoGAN on the right with training step.
  ALT Text: Two sets of line graphs tracking model performance over time. The left graph (our model) and right graph (ProteoGAN) both plot various metrics on the y-axis against parameter updates on the x-axis. Metrics include MMD, MRR, Generator Loss, and Discriminator Loss. The curves show the convergence behavior of the models during training.

- Fig. S10 caption: The influence of one-stage model(left) and two-stage model (right) for small datasets. In the one-stage, the input of model includes the GO annotations while the input of two-stage model is latent representations of protein sequences.
  ALT Text: Four line graphs comparing model performance on small datasets. The top row shows results for Malate dehydrogenase, and the bottom row shows results for Lysozyme. The left column displays the "one-stage model" performance, and the right column displays the "two-stage model" performance. The graphs track metrics like MMD, MRR, and Loss over parameter updates.

- Fig. S11 caption: t-SNE visualization from natural protein and generated protein with five different functions. a. Drug binding and transmembrane transporter activity. b. Catalytic activity, cofactor binding, and anion binding. c. Small molecule binding, signaling receptor activity, catalytic activity, acting on a protein and kinase activity. d. Ion transmembrane transporter activity and cation binding. e. DNA binding and RNA binding.
  ALT Text: Five scatter plots (labeled a through e) generated using t-SNE dimensionality reduction. Each plot shows clusters of data points representing natural and generated proteins, color-coded to distinguish between them. The clusters demonstrate the distribution overlap between generated and natural sequences for specific protein functions.

- Fig. S12 caption: The influence of the dimension of latent representation r and hyperparameter β on MRR and MMD. a. The MRR with different r values versus training steps; b. The MMD with different r values versus training steps; c. The MRR with different β values versus training steps; d. The MMD with different β values versus training steps.
  ALT Text: Four line charts analyzing hyperparameter sensitivity. Charts (a) and (b) show the trends of MRR and MMD respectively for different latent dimensions ($r = 10, 320, 640$) over training steps. Charts (c) and (d) show the trends of MRR and MMD for different β values (10, 100, 175, 200, 300) over training steps.

- Fig. S13 caption: a. The attention map for the natural lysozyme above and the generated lysozyme below. A larger value indicates that the amino acid residue at a position on the x-axis is in contact with the amino acid residue at a position on the y-axis. b. The lDDT value for each position: the natural lysozyme above and the generated lysozyme below. c. The results of the conservation analysis based on MSA for one natural lysozyme and one generated lysozyme, highlighting different gene ontology terms. d. Detailed 3D structure predictions by AlphaFold2, with the natural structure above and the generated prediction below.
  ALT Text: A composite figure comparing natural and generated lysozyme. Column (a) shows contact heatmaps. Column (b) shows line graphs of lDDT scores per position. Column (c) displays sequence logos with colored bars indicating conservation. Column (d) shows 3D ribbon diagrams of the protein structures. In all columns, the top image represents the natural protein and the bottom image represents the generated protein.

- Fig. S14 caption: Contact map with 3D structure input (left) and attention map with protein sequence input.
  ALT Text: Two heatmaps side-by-side. The left heatmap is a Contact Map derived from 3D

structure input, showing residue interactions on a dark background with yellow highlights. The right heatmap is an Attention Map derived from protein sequence input, showing a similar diagonal pattern of interactions on a white background with orange/yellow gradients.
